# Protein-protein interactions in neurodegenerative diseases: A conspiracy theory

**DOI:** 10.1371/journal.pcbi.1008267

**Published:** 2020-10-13

**Authors:** Travis B. Thompson, Pavanjit Chaggar, Ellen Kuhl, Alain Goriely, for the Alzheimer’s Disease Neuroimaging Initiative

**Affiliations:** 1 Mathematical Institute, University of Oxford, Oxford, United Kingdom; 2 Living Matter Laboratory, Stanford University, Stanford, California, USA; King’s College London, UNITED KINGDOM

## Abstract

Neurodegenerative diseases such as Alzheimer’s or Parkinson’s are associated with the prion-like propagation and aggregation of toxic proteins. A long standing hypothesis that amyloid-beta drives Alzheimer’s disease has proven the subject of contemporary controversy; leading to new research in both the role of tau protein and its interaction with amyloid-beta. Conversely, recent work in mathematical modeling has demonstrated the relevance of nonlinear reaction-diffusion type equations to capture essential features of the disease. Such approaches have been further simplified, to network-based models, and offer researchers a powerful set of computationally tractable tools with which to investigate neurodegenerative disease dynamics. Here, we propose a novel, coupled network-based model for a two-protein system that includes an enzymatic interaction term alongside a simple model of aggregate transneuronal damage. We apply this theoretical model to test the possible interactions between tau proteins and amyloid-beta and study the resulting coupled behavior between toxic protein clearance and proteopathic phenomenology. Our analysis reveals ways in which amyloid-beta and tau proteins may conspire with each other to enhance the nucleation and propagation of different diseases, thus shedding new light on the importance of protein clearance and protein interaction mechanisms in prion-like models of neurodegenerative disease.

## Author summary

In 1906 Dr. Alois Alzheimer presented the case of Ms. Auguste Deter; her symptoms would help to define Alzheimer’s disease (AD). Over a century later, with an aging world population, AD is at the fore of global neurodegenerative disease research. Previously, toxic amyloid-beta protein (A*β*) was thought to be the primary driver of AD development. Recent research suggests that another protein, tau, plays a fundamental role. Toxic tau protein contributes to cognitive decline and appears to interact with toxic A*β*; research suggests that toxic A*β* may further increase the effects of toxic tau. Theoretical mathematical models are an important part of neurodegenerative disease research. Such models: enable extensible computational exploration; illuminate emergent behavior; and reduce research costs. We have developed a novel, theoretical mathematical model of two interacting species of proteins within the brain. We analyze the mathematical model and demonstrate a computational implementation in the context of A*β*-tau interaction in the brain. Our model clearly suggests that: the removal rate of toxic protein plays a critical role in AD; and the A*β*-tau ‘conspiracy theory’ is a nuanced, and exciting path forward for Alzheimer’s disease research.

## Introduction

Neurodegenerative diseases such as Alzheimer’s (AD) or Parkinson’s (AD) are associated with the propagation and aggregation of toxic proteins. In the case of AD, it was Alzheimer himself who showed the importance of both amyloid-*β* (A*β*) plaques and tau-protein (*τ*P) neurofibrillary tangles (NFT) in what he called the “disease of forgetfulness” [1, 2]. These two proteins are very different. A*β* forms extracellular aggregates and plaques whereas *τ*P are intracellular proteins involved in the stabilization of axons by cross-linking microtubules that can form large disorganized tangles [3, 4]. Since the early 90’s, when it was first formulated, the “amyloid cascade hypothesis” has dominated the search for cures and treatments [5, 6]. According to this hypothesis, an imbalance between production and clearance of A*β*42 and other A*β* peptides is not only an early indicator of the disease but the causing factor for its initiation, progression, and pathogenesis [7]. However, the repeated failures of large clinical trials focussing on the reduction of A*β* plaques has led many researchers to question the amyloid hypothesis and argue for the possible importance of other mechanisms.

One obvious alternative is that *τ*P plays a more prominent role than the amyloid hypothesis suggests. The *τ*P are usually considered as secondary agents in the disease despite the fact that (1) other *τ*P-related diseases (tauopathies), such as frontotemporal lobar degeneration, are mostly dominated by *τ*P spreading [8]; (2) brain atrophy in AD is directly correlated with large concentrations of NFT [9, 10]; (3) *τ*P distribution determines disease staging [11]; (4) lowering *τ*P levels prevent neuronal loss [12]; (5) *τ*P reduces neural activity and is the main factor associated with cognitive decline [13]. These findings may explain the relative lack of clinical improvements after A*β* suppression and the debate between the relative importance of A*β* proteopathy and *τ*P tauopathy in AD [14]. Furthermore, the similarity in mechanism and progression between prion diseases [15] and classical neurodegenerative diseases led to the formulation of the “prion-like hypothesis” [16, 17, 18, 19, 20, 21] stating that all these protein-related degenerative diseases are characterized by the progressive spreading and autocatalytic amplification of abnormal proteinaceous assemblies through axonal pathways [22].

Since so many cellular mechanisms are poorly understood in vivo, the relative importance of different groups of toxic proteins and their possible interactions have not been established. In particular, both *τ*P and A*β* depend upon and modify the cellular environment [16]. Yet, in recent years a number of studies have linked these two anomalous proteins [23] and raised the possibility that protein-protein interactions in neurodegenerative diseases are a key to understanding both spreading and toxicity [24, 25]. According to Walker, for AD “the amyloid-*β*-*τ* nexus is central to disease-specific diagnosis, prevention and treatment” [14].

Specifically, the following crucial observations have been made in AD: (1) tangles in the cortex rarely occur without A*β* plaques [12]; (2) the presence of A*β* plaques accelerates both the formation of *τ*P aggregates [26] and the interneuronal transfer [27] of *τ*P; (3) the presence of *τ*P induces blood vessel abnormalities [28] and induces neuroinflammation through micro- and astro-glial activation [29]; (4) the presence of A*β* can induce the hyperphosphorylation of *τ*P and the creation of toxic *τ*P seeds by disturbing cell signaling via oxidative stress or through plaque-associated cells (such as microglia) or molecules [30, 31, 32, 33]; (5) A*β* and toxic *τ*P target different cellular components, and doing so amplify each other’s toxic effects [23]; (6) *τ*P mediates A*β* toxicity as a reduction of *τ*P prevents A*β*-induced defects in axonal transport [23]; (7) perhaps more anecdotal, it has also been argued that the lack of clear evidence of dementia in non-human primates, despite the presence of A*β* plaques, could be due to a difference in A*β*-*τ* interactions in these species [34].

From these observation, we extract three crucial modes of interaction:

**M1**: The seeding of new toxic *τ*P is enhanced by the presence of A*β*.
**M2**: The toxicity of A*β* depends on the presence of *τ*P.
**M3**: A*β* and *τ*P enhance each other’s toxicity.

Here, our goal is twofold: first to develop modeling and computational tools to study protein-protein interactions at the brain-organ level and second to test the relative effect of these interactions by direct simulation. Typical approaches for organ-size simulation of dementia progression [35] take the form of either continuous models formulated in terms of anisotropic reaction-diffusion equations [36, 37, 38], or discrete systems on the brain’s connectome network. The discrete approach can be further divided into pure-diffusion linear models [39, 40, 41, 42, 43], probabilistic models [44, 45, 46], or deterministic models [47, 48].

A primary result, of interest to the computational biology community, for the current work will be to show: that non-trivial interactions between A*β* and *τ*P can be realized with relatively simple deterministic models and couplings; and that these interactions can lead to effects with physiological interpretations in neurological disease modeling. Moreover, the mathematical analysis will highlight that clearance mechanisms play a key role in destabilizing the system towards proteopathy. We will therefore select the simplest possible, deterministic, protein kinetic model, including a bulk clearance term, that allows for the expression of both a healthy and toxic regime for a single protein; the heterodimer model [49, 50]. One such system will be defined for A*β*, one for *τ*P and the two heterodimer systems are coupled with a single balanced interaction term. We augment the model by adding an stand-alone, first order equation for damage evolution; this equation expresses the deleterious effects of A*β*, *τ*P and their interactions. Our general approach, following [48] is to study some of the key properties of this continuous model before discretizing it on a network and solving it numerically on the brain’s connectome graph.

## Results

### Network model dynamics

We have established the properties of our system of equations in the homogeneous case and in one-dimension (Methods, A network mathematical model). The study has lead to the identification of two fundamental disease propagation modes depending on the parameters: the primary tauopathy where toxic *τ*P states can exist independently from the A*β* concentration, but are enhanced by its presence; and the secondary tauopathy where the presence of toxic *τ*P is slaved to the existence of toxic A*β*. We can use this analysis as a guide to the simulation of the full network equation. Eqs (8)–(12) were discretized on the reference connectome [59] (Methods, A network mathematical model) using CVODE as part of the SUNDIALS nonlinear ODE solver library [63] in addition to KLU [64] as part of the SuiteSparse [65] linear algebra library. Snapshots of the dynamics are shown in subsequent figures, but full videos can be found in the supplementary material.

As a way to systematically test the validity of our computational platform, we have performed two main tests. First, we reproduced the homogeneous states in the full network and second, we reproduce the transition between homogeneous states. Both tests are detailed in S1 Appendix in addition to a discussion regarding a choice of hypothetical, non-clinical parameters for illustration purposes; S1 Appendix contains a full discussion on the specific numeric values of the stationary states corresponding to the choices of of Table 1.

**Table 1:** Primary tauopathy model parameters.

A*β* system parameters	*τ*P system parameters
*a*_0_ = 0.75	*b*_0_ = 0.5
*a*_1_ = 1.0	*b*_1_ = 1.0
*a*_2_ = 1.0	*b*_2_ = 1.0
${\tilde{a}}_{1}=0.6$	${\tilde{b}}_{1}=0.4$
Coupling parameter:	*b*_3_ = 1.0

#### Front dynamics on networks

Propagating front solutions for the system of partial differential Eq (3) were considered, via linearization around the healthy state and reduction to one spatial dimension, (c.f. Methods, Front propagation), Propagating fronts represent fundamental modes of disease pathology dynamics that can also be realized by the network model of (8)–(12) as we now demonstrate. We consider two different network for front propagation. First, a three-dimensional regular cubic lattice with *n*_*x*_ = 30 nodes in the *x*-direction *n*_*y*_ = 6 nodes in the *y*-direction and *n*_*z*_ = 3 nodes in the *z*-direction, spaced equally at unit length. Second, we use the physiological brain connectome domain discussed in the Methods section (A network mathematical model), but we choose initial conditions on two sides of the brain to illustrate the front dynamics. In the next section we will consider the same domain but with realistic initial conditions.

The first example is that of primary tauopathy corresponding to the parameters of Table 1. Formulas for the steady states, for primary tauopathy, are listed in the Methods (An Analysis of the continuous model, Stationary points) section.

#### Primary tauopathy

#### Primary tauopathy, synthetic domain

**Fig 1:**
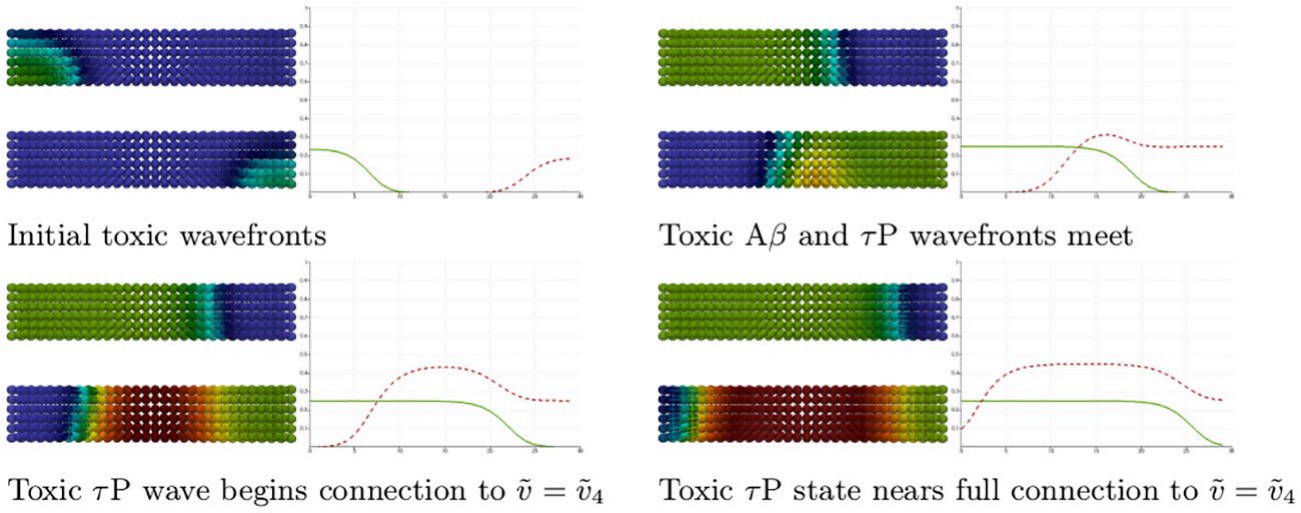
Front propagation in primary tauopathy; synthetic rectangular domain. Each subfigure consists of a toxic A*β* concentration distribution (top left), toxic *τ*P concentration distribution (bottom left) and a plot (solid line: A*β*, dashed line: *τ*P) of the concentration level on the *x*−axis. Dark blue indicates the minimum concentration of *c* = 0.0 while bright red indicates the maximum of *c* = 0.5. See the Methods section (Front propagation) for a comparison to theory. (See also: supplementary S1 Video).

We set all nodes to the healthy state $\left(u,\tilde{u},v,\tilde{v}\right)=\left(0.75,0,0.5,0\right)$ and perturb the initial condition of the left-hand nodes 0 ≤ *x* ≤ 4 by adding a 5% concentration ($\tilde{u}=0.05$) of toxic A*β*. We perturb the initial condition of the right-hand nodes 25 ≤ *x* ≤ 29 by adding a 5% concentration ($\tilde{v}=0.05$) of toxic *τ*P. As expected, we see the toxic A*β* concentration achieve the theoretical maximum, permitted by the parameters, of $\tilde{u}=0.25$ while toxic *τ*P first achieves the maximum associated with $\tilde{v}=v_{3}=0.25$ and, upon mixing with A*β*, achieves the fully toxic state value $\tilde{v}=v_{4}=0.45$. The color scale of Fig 1 was chosen to accentuate the interaction.

#### Primary tauopathy, brain connectome

**Fig 2:**
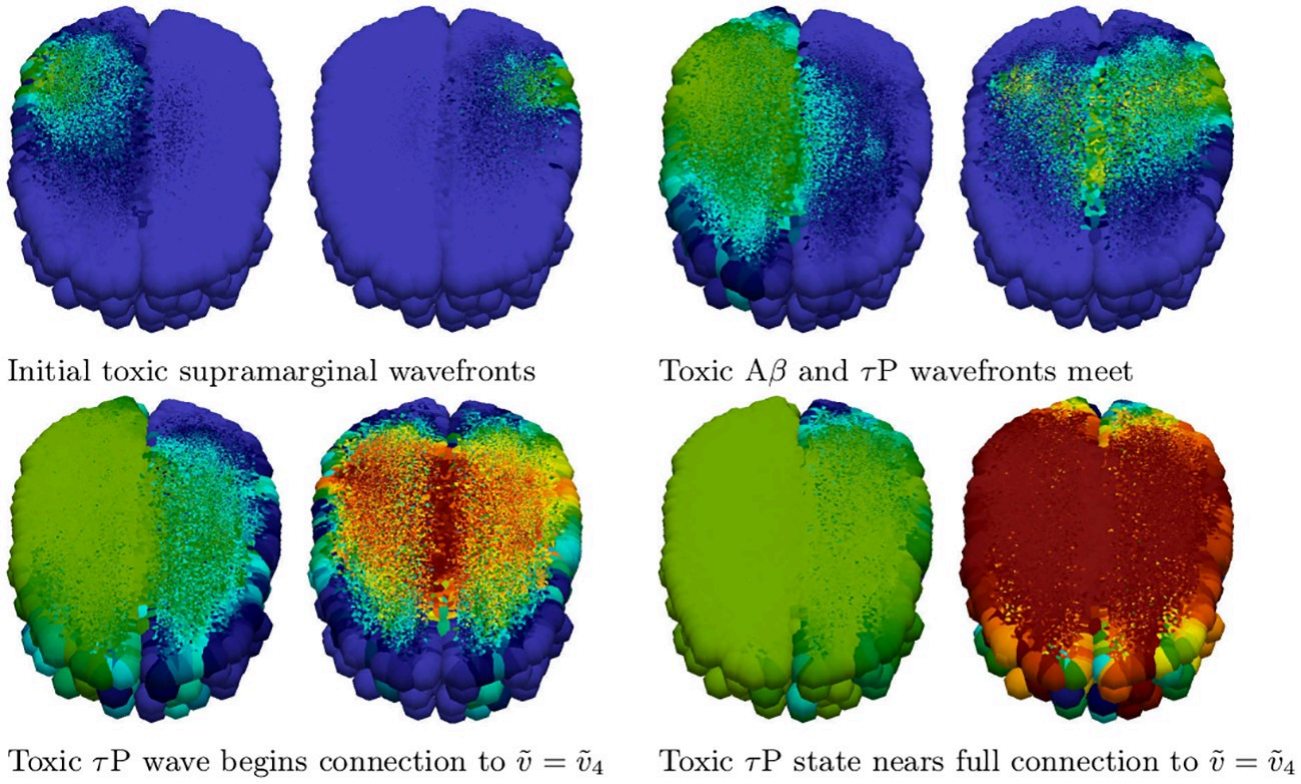
Front propagation in primary tauopathy; brain connectome. Each subfigure consists of a toxic A*β* concentration distribution (subfigure left) besides a toxic *τ*P concentration distribution (subfigure right). Dark blue indicates the minimum concentration of *c* = 0.0 while bright red indicates the maximum of *c* = 0.5. (See also: supplementary S2 Video and supplementary fle S2 Data).

Simulation of disease front propagation was then carried out using the physiological connectome (Methods, A network mathematical model). The seeding sites selected for toxic A*β* and toxic *τ*P are the right supramarginal gyrus and left supramarginal gyrus respectively; these seeding sites provide a direct analogy, when the brain connectome is viewed from the frontal lobe, with Fig 1. Fig 2 depicts time instances qualitatively reflecting, in one-to-one correspondence, the stages of the synthetic domain computation of Fig 1. A horizontal slice, at the plane of the supramarginal gyri, of the brain connectome is used to maximally expose the front propagation dynamics. The impact of brain connectome cross-connectivity is evident in the stages depicted in Fig 2. In particular, when the A*β* and *τ*P wavefronts first meet they do so in several locations. This is due to the left-right hemispheric connectivity; both direct nodal connectivity and vis-a-vis propagation in the coronal plane.

#### Secondary tauopathy

The parameters for the at-risk secondary tauopathy patient are those of Table 1 with two exceptions; first, as usual for secondary tauopathy, we take *b*_2_ = 0.75 and second we take *b*_3_ = 3.0. We have increased *b*_3_ to facilitate the comparison with front propagation theory, for secondary tauopathy, discussed in the Methods section.

#### Secondary tauopathy, synthetic domain

Secondary tauopathy consists of all stationary states except for the toxic *τ*P–healthy A*β* state; i.e. $\left(u_{3},{\tilde{u}}_{3},v_{3},{\tilde{v}}_{3}\right)$ is not included. The stationary point $\left(u_{4},{\tilde{u}}_{4},v_{4},{\tilde{v}}_{4}\right)$ depends on *b*_3_; with the parameters above we have

$$\begin{array}{c} \left(u_{4},{\tilde{u}}_{4},v_{4},{\tilde{v}}_{4}\right)=\left(0.6,0.25,\frac{1.6}{b_{3}+3},\frac{5b_{3}-1}{4b_{3}+12}\right)=\left(0.6,0.25,0.2\bar{6},0.58\bar{3}\right), \end{array}$$ (1)

while the other two secondary tauopathy stationary points, c.f. (14) and (15), coincide with their values for primary tauopathy. The initial value at all nodes are first set to the healthy state. A 5% perturbation in concentration is then added to the toxic A*β* initial value for the nodes 0 ≤ *x* ≤ 4 and a perturbation of 1 × 10^−9^%, i.e. 1 × 10^−11^, is added to the toxic *τ*P initial value for the nodes 0 ≤ *x* ≤ 14.

**Fig 3:**
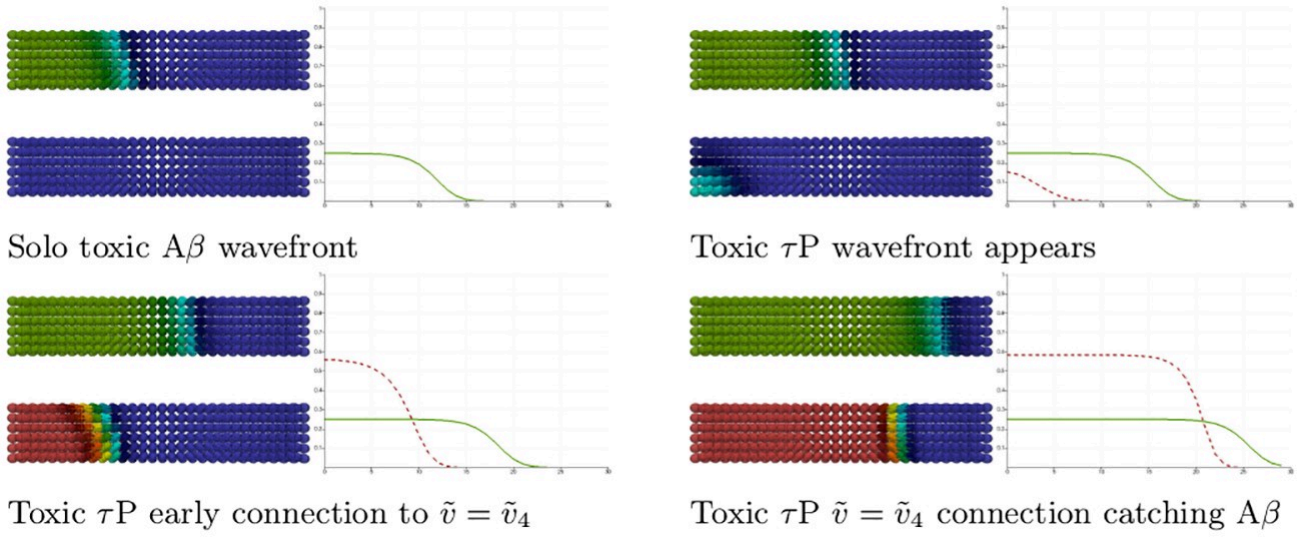
Front propagation in secondary tauopathy; rectangular domain. Each subfigure consists of a toxic A*β* concentration distribution (top left), toxic *τ*P concentration distribution (bottom left) and a plot (solid line: A*β*, dashed line: *τ*P) of the concentration level on the *x*−axis. Dark blue indicates the minimum concentration of *c* = 0.0 while bright red indicates the maximum of *c* = 0.5 for toxic A*β* and $c=0.58\bar{3}=7/12$ for toxic *τ*P. See the Methods section (Front propagation) for a comparison to theory. (See also: supplementary S3 Video).

As expected: the initial toxic A*β* wavefront achieves its theoretical maximum of $\tilde{u}=0.25$; c.f. Fig 3 and the front propagation discussion, for secondary taopathy, in the Methods section. The toxic *τ*P wave takes on detectable concentration levels at the point when the A*β* wave reaches the halfway mark in the rectangular domain. The toxic *τ*P state connects, immediately, to the theoretical maximum of the toxic *τ*P–toxic A*β* stationary state value of ${\tilde{v}}_{4}=7/12$ and quickly proceeds to catch up to the A*β* wavefront.

**Fig 4:**
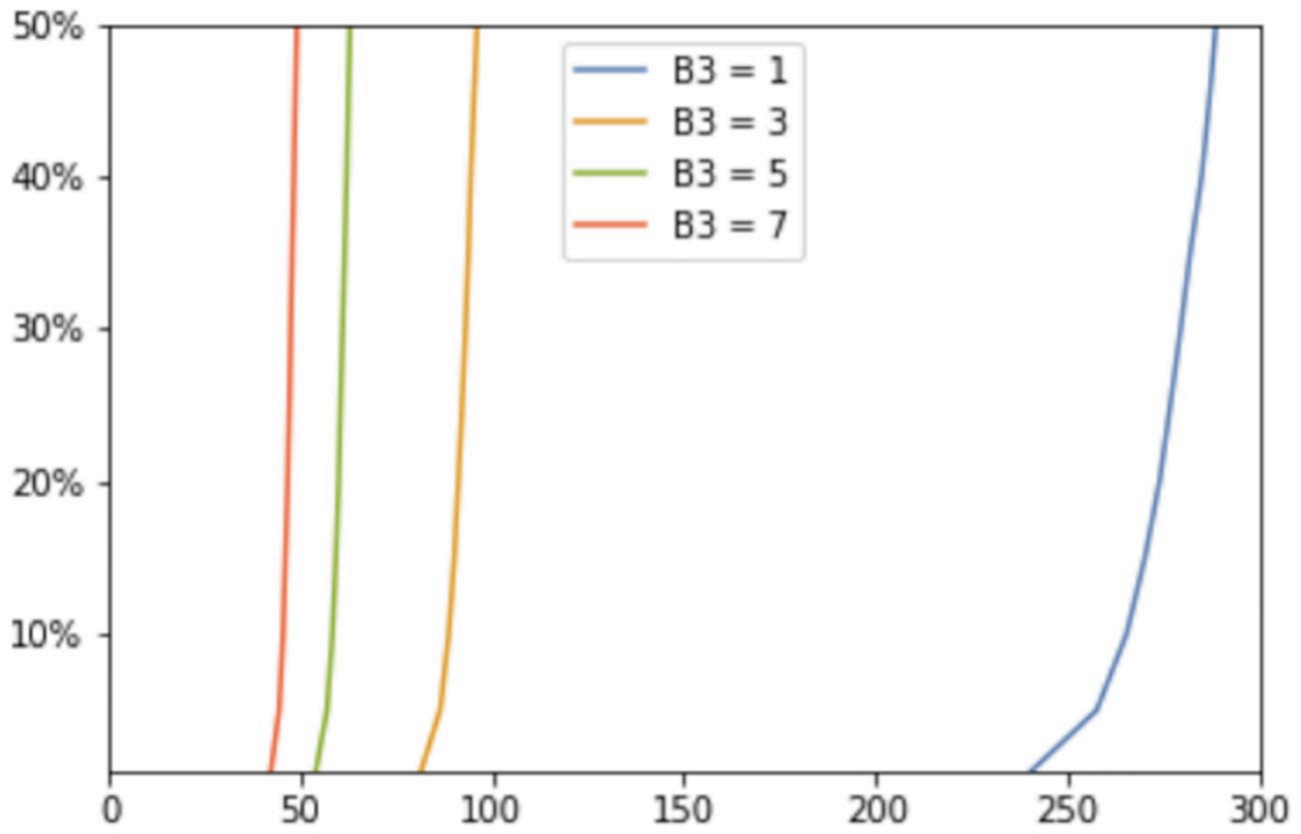
The onset effect due to *b*_3_ in secondary tauopathy. Saturation % (*y*-axis) vs Simulation time (*x*-axis).

We tested the time of appearance and saturation of the toxic *τ*P wave front as a function of the interaction parameter *b*_3_. Plots for four values of *b*_3_ are shown in Fig 4 where the y-axis signifies the maximal toxic *τ*P concentration obtained, over all nodes, with respect to the maximum concentration for that value of *b*_3_ (c.f. (1)). Fig 4 highlights the important, and patient-specific, role that *b*_3_ may play in further efforts to deploy (8)–(11) for the modeling of Alzheimer’s disease. In particular values of *b*_3_ ≈ 1 do lead to the development of tauopathy; however, this development emerges significantly later than for higher values of this interaction parameter. Clinically, such a value of *b*_3_ could correspond to a patient who, at the time of death, presents significant amyloid plaques but negligible, or undetectable, levels of neurofibrillary tau tangles.

#### Secondary tauopathy, brain connectome

**Fig 5:**
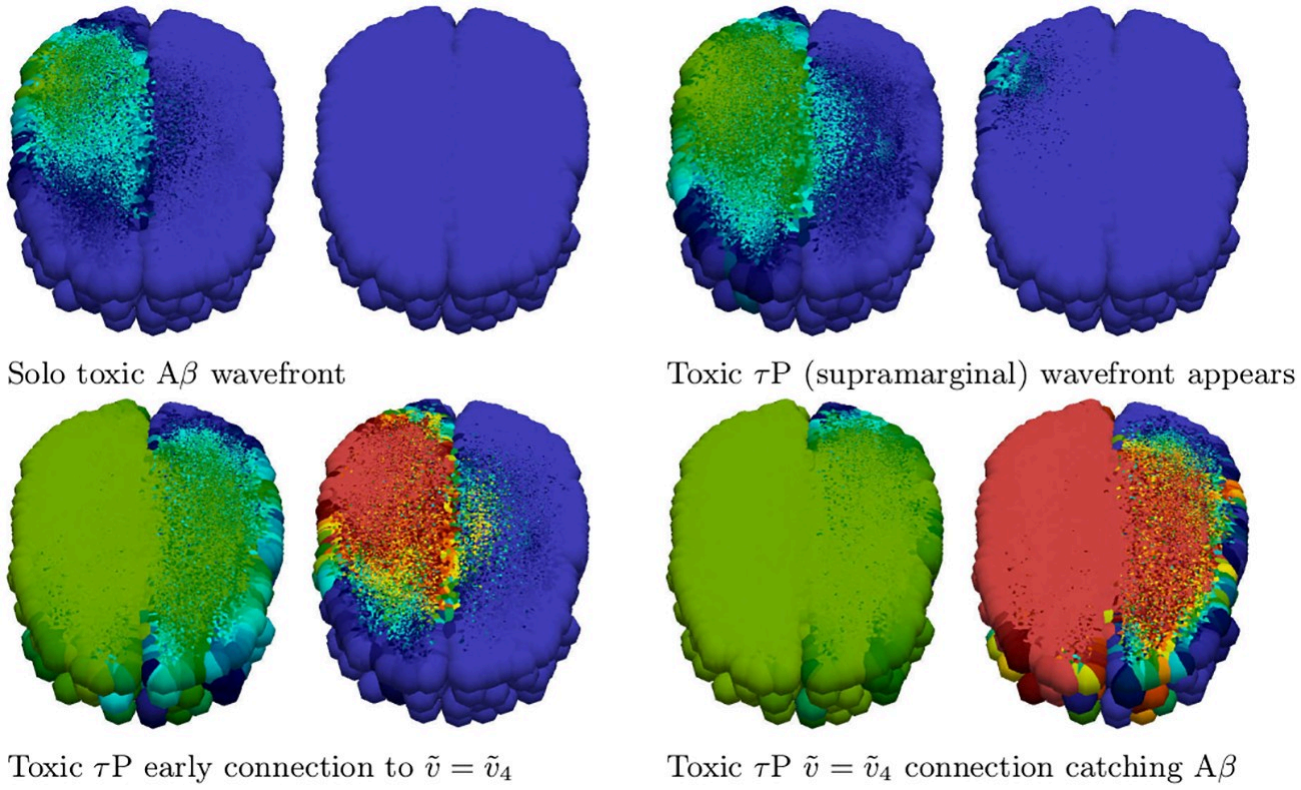
Front propagation in secondary tauopathy; brain connectome. Each subfigure consists of a toxic A*β* concentration distribution (subfigure left) besides a toxic *τ*P concentration distribution (subfigure right). Dark blue indicates the minimum concentration of *c* = 0.0 while bright red indicates the maximum of *c* = 0.5 for toxic A*β* and $c=0.58\bar{3}=7/12$ for toxic *τ*P. (See also: supplementary S4 Video and supplementary S2 Data).

We also simulated secondary tauopathy dynamics on the physiological brain connectome (Methods, A network mathematical model). A 5% toxic A*β* perturbation from the healthy state was seeded at the site of the left supramarginal gyrus; all nodes of the left hemisphere were then seeded with an additional 1 × 10^−9^% concentration of toxic *τ*P. Snapshots of the evolution is shown in Fig 5. As indicated above we have *b*_3_ = 3 for comparison with Fig 3 and the theory of propagating fronts as illustrated in the Methods section. A detail of particular interest is that, even though the entire left hemisphere was seeded uniformly with toxic *τ*P, the toxic *τ*P wave follows the same anisotropic infection pathway, from the left supramarginal gyrus, as the toxic A*β* front propagation. This implies that latent development of tauopathy, in this regime, is heavily influenced by A*β* pathology history.

### Application to neurodegenerative disease modeling

We have shown in the previous section that the overall phenomenology obtained from the dynamic evolution of the continuous model in one-dimension (Methods, A network mathematical model, Front Propagation) is recovered within the discrete network setting. We can therefore use the network model and our primary classification to study the interaction of proteins in the brain. Here, we apply (8)–(11) to a computational case inspired by Alzheimer’s disease. In particular we consider seeding sites, for toxic A*β* and toxic *τ*P, commensurate with [11, 66, 67, 47] Alzheimer’s disease staging. Alzheimer’s disease is a complex multiscale phenomena; a uniform parameter regime, throughout all brain regions, is unlikely to accurately reflect a patient’s real disease progression. Nevertheless, for this early investigation, we will first consider the simple uniform parameters, of the model’s primary and secondary tauopathy regimes, as discussed in the section on network model dynamics. In addition we briefly consider the evolution of the coupled neuronal damage term, given by (12), and the effect of the coefficients therein. We shall also select the diffusion constants, *ρ* of (7), to be unity for (8)–(11).

#### A simplified model of Alzheimer’s disease proteopathy

**Fig 6:**
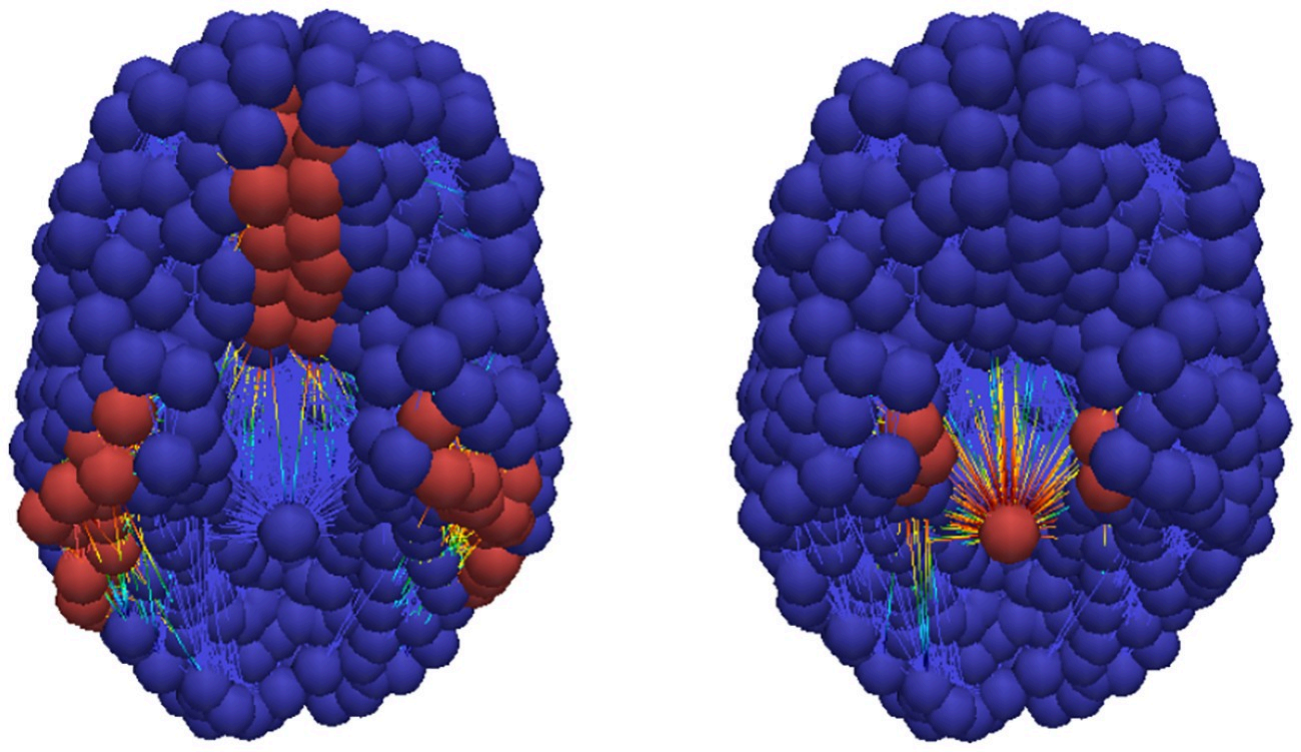
Simulated seeding sites for a model of Alzheimer’s disease. Toxic A*β* (left) and toxic *τ*P (right) seeding sites.

Alzheimer’s associated amyloid deposition begins [18, 47, 66, 67] in the temporobasal and frontomedial regions. Tau staging, in Alzheimer’s disease, follows the Braak tau pathway [11] and begins in the locus coeruleus and transentorhinal layer [18, 47, 67]. These seeding sites, used throughout this section, are shown in Fig 6. The temporobasal and frontomedial regions for toxic A*β* seeding are highlighted in red on the left while the locus coeruleus (in the brain stem) and transentorhinal associated regions, for toxic *τ*P staging, are highlighted red on the right.

**Fig 7:**
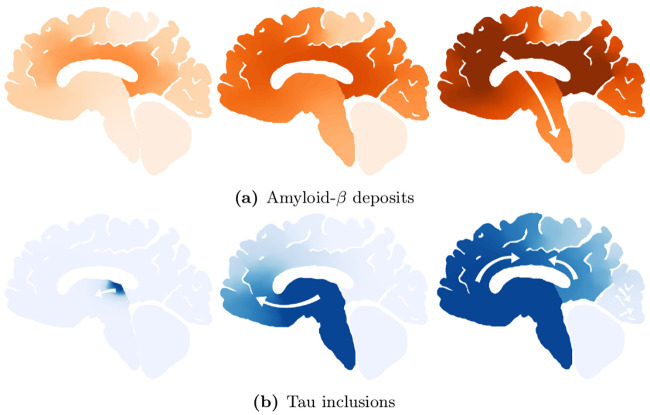
Characteristic progression of of A*β* and *τ*P lesions. 3-stage A*β* (top) progression and *τP* NFT (bottom) progression.

The regimes of primary and secondary tauopathy will first be considered on the whole brain connectome with globally-constant synthetic parameters. We will observe several characteristic traits of these modalities and also note the similarity between these pure states and to a qualitative three-stage progression [18] of protein lesions, typical of Alzheimer’s disease, as inferred from post-mortem analyses; the progression pattern is illustrated in Fig 7. In the subsection titled ‘a mixed model comparison to Alzheimer’s diseased patient data’ we consider the case of mixed regional modalities; i.e. a mixture of primary and secondary tauopathy connectome regions. We illustrate that the model can manifest canonical features of positron emission tomography (PET) SUVR intensities characteristic of Alzheimer’s disease (c.f. for instance [68, 69]). In particular: we will compare the results of a mixed-mode simulation with a cross sectional Alzheimer’s patient cohort dataset procured from the Alzheimer’s Disease Neuroimaging Initiative (ADNI) database.

#### Alzheimer’s disease, primary tauopathy

All nodes in the connectome were first set to the healthy, but susceptible, primary tauopathy patient state

$$\begin{array}{c} \left(u_{1},{\tilde{u}}_{1},v_{1},{\tilde{v}}_{1}\right)=\left(\frac{a_{0}}{a_{1}},0,\frac{b_{0}}{b_{1}},0\right)=\left(0.75,0,0.5,0\right). \end{array}$$

The temporobasal and frontomedial A*β* seeding sites, consisting of fifty-three nodes, were each seeded with a toxic amyloid concentration of 0.189%; thus the brain-wide toxic A*β* concentration represents a 1% concentration deviation from healthy. Similarly, the locus coeruleus and transentorhinal nodes were seeded with an aggregate perturbation of 1% toxic *τ*P.

**Fig 8:**
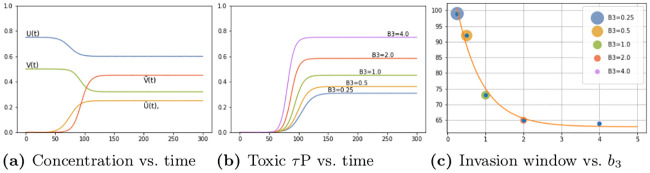
Protein-protein interaction in primary tauopathy.

Fig 8a shows the average brain-wide concentration for all four protein populations for the primary tauopathy patient (c.f. Table 1) with interaction term *b*_3_ = 1. As we observed previously, in (1), the value of *b*_3_ directly informs the saturation *τ*P concentration, of $\left(v,\tilde{v}\right)$, for the disease. Fig 8b shows the evolution of the toxic *τ*P burden for various *b*_3_. For each value of *b*_3_ the toxic *τ*P invasion window was computed as the difference in time between the appearance of a global 1% toxic *τ*P concentration to the simulation time where the maximum $\tilde{v}$ was reached. We performed a least squares fit and found that the invasion window, for primary tauopathy, decreases exponentially with an increase in coupling strength (*b*_3_) between toxic A*β* and toxic *τ*P. Fig 8c shows the result.

This result suggests that the dynamics of toxic protein evolution is highly sensitive to the coupling between A*β* and *τ*P: Toxic A*β* accelerates, in a nonlinear fashion, the way toxic *τ*P emerges across the brain. Acceleration of toxic *τ*P progression due to the presence of toxic A*β* has also been observed in mouse models of Alzheimer’s disease [27]. Consulting longitudinal tau PET studies, in combination with amyloid-beta data from a public database, could provide an estimation of *b*_3_ in the primary tauopathy model.

**Fig 9:**
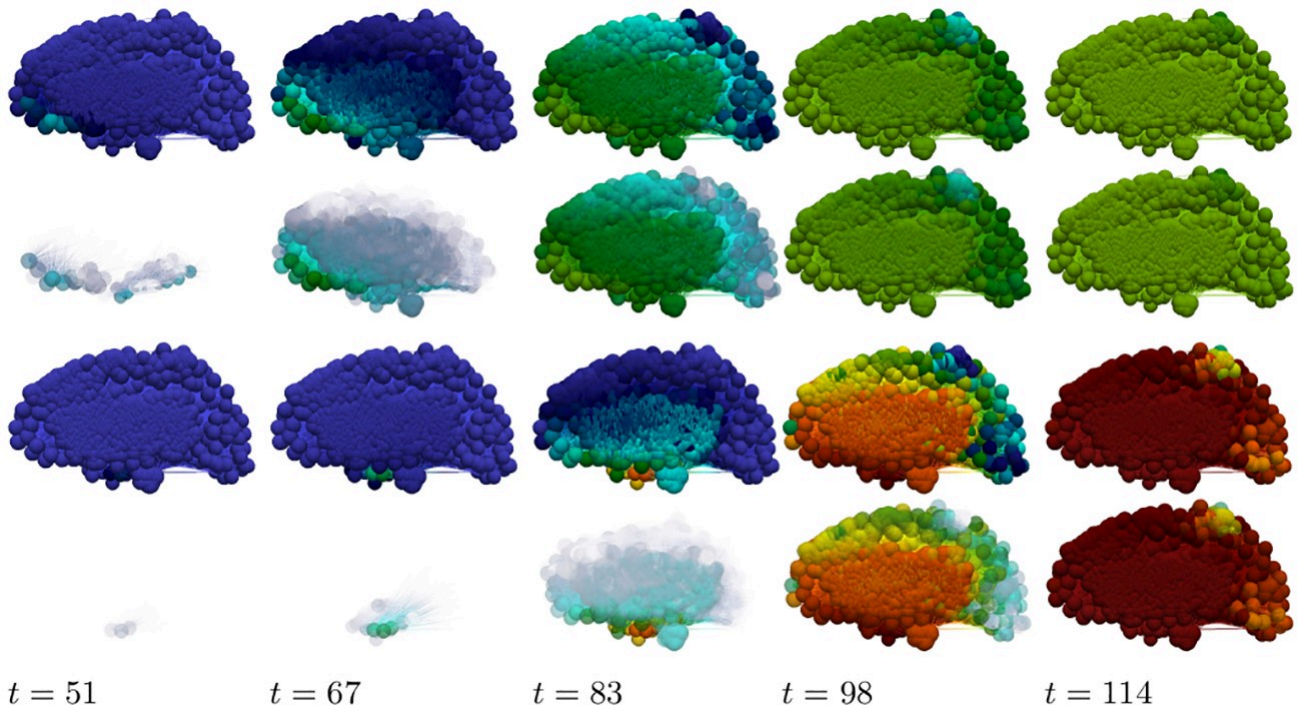
Toxic proteopathy progression dynamics in the primary tauopathy patient. Toxic A*β* (top row) and opacity exaggerated toxic A*β* progression (second row); Toxic *τ*P (third row) and opacity exaggerated toxic *τ*P progression (last row). Color scale is identical to Fig 1. (See also: supplementary S5 Video, supplementary S6 Video and supplementary S2 Data).

The toxic load progression of the susceptible primary tauopathy patient is shown in Fig 9 at five equidistant time points throughout the invasion window. To facilitate a comparison with Fig 7: a sagittal view of the progression, of each toxic agent, is presented; directly below is an opacity-exaggerated view wherein regional opacity is proportional to the agent’s regional toxic load. Comparison with Fig 7 suggests reasonable qualitative agreement; thus warranting further study of physically relevant parameters with a view towards real clinical applications.

#### Alzheimer’s disease, secondary tauopathy

All nodes were set to the healthy, but susceptible, patient state corresponding to the susceptible secondary tauopathy patient parameters (Table 1 with *b*_2_ = 0.75). In addition, for a baseline secondary tauopathy case, we follow the secondary tauopathy approach discussed previously and select the interaction parameter of *b*_3_ = 3.0; the fully invaded secondary tauopathy state values are therefore (1). Seeding patterns for both A*β* and *τ*P are identical to the case of primary tauopathy discussed above.

**Fig 10:**
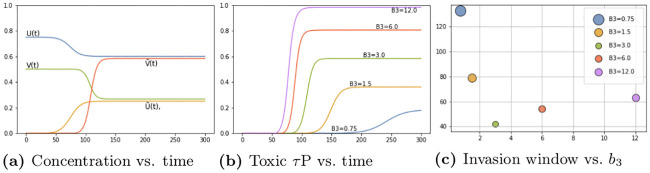
Protein-protein interaction in secondary tauopathy.

Fig 10a shows the average brain-wide concentration for all four protein populations of the secondary tauopathy patient with baseline interaction term *b*_3_ = 3. As in the case of primary tauopathy we investigate the effect of *b*_3_ on toxic load and invasion window by considering a value range four times smaller to four times larger than the baseline *b*_3_ = 3 case. Toxic load curves are shown in Fig 10b while invasion windows are shown in Fig 10c.

**Fig 11:**
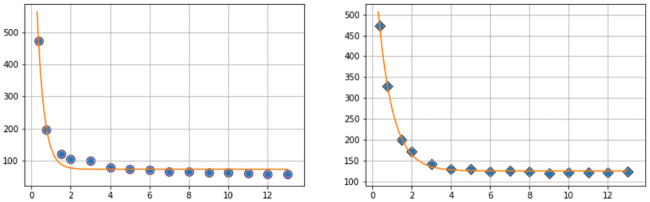
Prodromal window variations with *b*_3_, secondary tauopathy. Invasion starting (left) and ending (right) time vs. *b*_3_.

Interestingly, we see distinct differences in comparison with the primary tauopathy case (c.f. Fig 8a–8c). More specifically, in primary tauopathy it is evident (Fig 8b) that the disease onset is only slightly affected by varying the interaction parameter *b*_3_; for secondary tauopathy, in contrast, *b*_3_ has a profound effect on disease onset latency. Moreover, the invasion window variation with *b*_3_ for secondary tauopathy is more complex than that of primary tauopathy. Fig 10c shows that the invasion window duration initially decreases exponentially with *b*_3_ but then appears to increase logarithmically for *b*_3_ ≥ 3. Analyzing the invasion window start time and end time separately shows a clear, but separate, exponential decay pattern versus *b*_3_. Fig 11 shows the least-squares exponential fit to the invasion start and end times.

**Fig 12:**
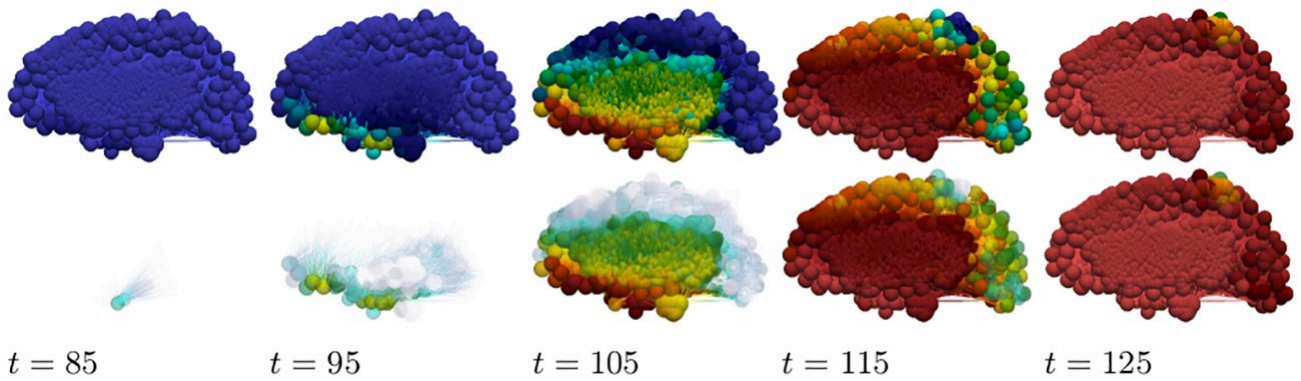
Toxic *τ*P progression dynamics in the secondary tauopathy patient. Toxic *τ*P (first row) and opacity exaggerated toxic *τ*P progression (second row). Color scale is identical to the *τ*P case of Fig 3. (See also: supplementary S5 Video, supplementary S7 Video and supplementary S2 Data).

As in the primary tauopathy case we now consider characteristic toxic load progression for secondary tauopathy. The A*β* progression is identical to that shown in Fig 9 (top two rows). This is expected as only the *τ*P portion of the system has been modified with respect to the primary tauopathy regime (S1 Appendix). The *τ*P secondary tauopathy progression is shown, in Fig 12, at equally spaced simulation times through the invasion window. Qualitatively, the progression of secondary tauopathy also reflects the characteristic post-mortem progression of Fig 7.

#### A mixed model comparison to Alzheimer’s diseased patient data

Thus far, we have considered, respectively, the general features of the modalities of primary versus secondary tauopathy; illustrated with synthetic, globally constant parameters. We have observed several interesting facets of these two disease states. For instance, regions in a state of primary tauopathy can develop A*β* and *τ*P proteopathy separately; the A*β* interaction parameter, *b*_3_, does not alter the onset of tauopathy but does modulate the regional concentration. Conversely, in secondary tauopathy the presence of A*β* pathology is necessary for *τ*P pathology and the interaction parameter, *b*_3_, modulates both the latency and the intensity of the regional pathology. We have also seen that proteopathy progression in pure models, e.g. where all regions have the same primary or secondary tauopathy parameters, bears a notable resemblance (Fig 7) to post-mortem progression of protein lesions [18]. However, PET imaging studies of A*β* and *τ*P radiotracer uptake tell a more nuanced story. For instance, in Alzheimer’s disease the distribution of ([^18^F]flortaucipir and [^18^F]THK-5117, among others) PET-*τ*P SUVR intensities are distinctly biased [68, 69] towards the temporal and parietal regions of the brain; a feature that we do not see in Fig 9 or Fig 12.

In order to demonstrate that the model of (8)–(11) can reproduce salient features of A*β* and *τ*P SUVR uptake in patients diagnosed with Alzheimer’s disease: we now compare a mixed-modality simulation with a cross-sectional study of Alzheimer’s disease patient data. Sample data for model comparison was procured from the the Alzheimer’s Disease Neuroimaging Initiative (ADNI) database. We first queried the ADNI database to locate AD-diagnosed subjects between the age of 70 and 90 who had at least one *τ*P PET (18F-AV1451, flortaucipir) scan. The returned results consisted of 41 patients. These initial patient candidate IDs were then checked for a structural T1 weighted MRI within a maximum of one year of a tau PET scan; patients without any sMRI, those with a poor quality sMRI, or those without an sMRI within a year of the tau PET scan were discarded. The resulting cohort consisted of 38 patients (25 male and 13 female) with mean age 78.4, a standard deviation of 5.14 years, and a male-to-female ratio of 1.92. Patients who met the age, *τ*P PET scan, and acceptable quality sMRI within one year of the PET scan were not abundant in the ADNI database. Due to this the *τ*P group was selected, first, to maximise the number of candidates in the group.

The ADNI database contains quite a generous number of patients with A*β* (18F-AV45, florbetapir) PET scans. We next queried the database to locate AD-diagnosed subjects between 70 and 90 who had an A*β* (18F-AV45) PET scan in addition to a structural T1 weighted MRI within one year. The result of this search was in excess of 100 unique patient IDs; from these results we selected an initial candidate group of 82 unique patients IDs. The 82-candidate group was further pruned to create a list of 48 subjects whose age and sex characteristics closely resembled that of the *τ*P PET group. Finally, the 48 candidate A*β* PET group was narrowed down: first, subjects with an unacceptable or low-resolution sMRI were removed. We then removed the minimum number of candidates required to provide as close a match as possible to the mean age, standard deviation and male-to-female ratio of the *τ*P PET group. The resulting AD cohort for AB consisted of 42 patients with mean age 78.4, a standard deviation of 5.1 years, and a male-to-female ratio of 2.0. The two groups are succinctly summarised in Table 2.

**Table 2:** Adni patient group statistics.

	18F-AV1451 *τ*P PET group	18F-AV45 A*β* PET group
Total patient count	38	42
Male patients	25	28
Female patients	13	14
Mean age ± SD, years	78.4 ± 5.14	78.4 ± 5.1

Patient data was then processed through a semi-automated, scripted software pipeline for general connectome-graph based imaging and analysis of clinical patient data. Each of the 160 patient images, the PET and sMRI scan for each patient, were first manually analysed using version 12 of the Statistical Parameteric Mapping [70] (SPM) software; the origin of the image was set to coincide with the anterior commissure. Next, the sMRI images for each patient were pre-processed for connectome-graph visualization. The SPM software was used, on each patient sMRI, to perform a unified segmentation procedure [70]. The unified segmentation procedure identifies grey matter, white matter, cerebrospinal fluid, skull, and exterior regions. Following this, the grey and white matter segmentations served as input for spatial normalisation using the DARTEL [71] toolbox; the outputs of which were a composite template for the A*β* patient group and a separate composite template for the *τ*P patient group. All patient grey matter images were then normalised to MNI-152 space using their group-specific optimised DARTEL template.

**Fig 13:**
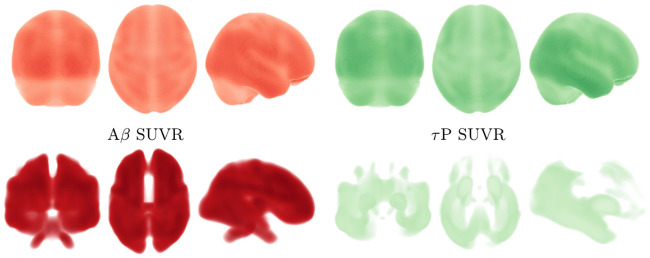
Skull-stripped, cross-sectional Alzheimer’s patient cohort SUVR intensity. Top row: averaged SUVR data is shown. Bottom row: top 30% of SUVR intensities are visible. For both rows: (left side) 18F-AV45 florbetapir A*β* radiotracer SUVR and (right side) 18F-AV-1451 flortaucipir *τ*P radiotracer SUVR. Darker colors correspond to higher SUVR values.

The next step of the pipeline is to treat the PET images for both the A*β* and *τ*P groups. This step relies on the fact that we have already manually relocated the origin of the PET images to the approximate visual location of the anterior commissure as mentioned above. The first new step for this portion of the pipeline is to use the SPM software to co-register the PET images to their sMRI counterparts. This co-registration step is the genesis of the original data procurement requirement that an sMRI scan is conducted no later than one year beyond the PET image acquisition date. The coregistered PET images are then spatially normalised using the DARTEL template and corresponding subject deformation fields derived from the sMRI pipeline (c.f. above). Finally, SUVR values were computed, using SPM, by means of a whole-cerebellar reference region; the skull was then stripped. A voxel-wise mean, across all subjects, was taken to produce a representative SUVR map of both AD cohorts. This completes the first portion of the connectome-graph based imaging analysis pipeline; the result of this step, for both the A*β* and *τ*P group, is shown in the top row of Fig 13.

**Fig 14:**
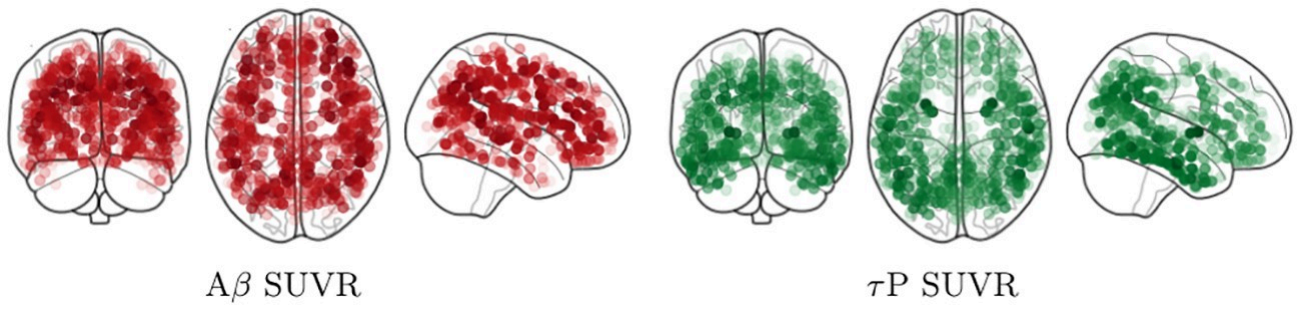
A connectome-graph view of the normalized patient SUVR data. The (left side) 18F-AV45 florbetapir A*β* radiotracer SUVR and (right side) 18F-AV-1451 flortaucipir *τ*P radiotracer SUVR. Highest 30% of connectome regional values are visible. Darker colors correspond to higher SUVR values.

The averaged SUVR data of Fig 13 (top row) reports a general view of uptake across the whole brain. In order to visualize significant features of the data: the skull-stripped SUVR image volumes, in NIfTI file format, are visualized using Paraview vis-a-vis the NIfTI Paraview plugin. The volume opacity then set so that the top 30% of the SUVR intensity range in the data is visible; c.f. Fig 13, middle row. Doing so: we immediately see notable features of significance reported in previous radiotracer studies; in particular the familiar [68, 69] temporal and parietal dominance of the *τ*P radiotracer uptake distribution (Fig 13, middle right) are visible. In order to compare simulation results to the patient data of Fig 13 we now employ a connectome-graph data visualization software process. This portion of the general pipeline uses functionality from both SPM and the Nilearn [72] Python library. Regional masks were produced using the Lausanne multiresolution atlas [73] parcellation to the MNI ICBM 152 non-linear 6th generation symmetric volume [74]; generating over 1000 distinct masks. The mask volumes were then applied to isolate the SUVR values for each mask in the parcellation and a regional average SUVR was computed. The computed values were then normalized to lie in the interval [0, 1] by dividing all regional SUVR averages by the global maximum average SUVR intensity; the normalized values, along with the MNI-space coordinates of the region’s centroid were recorded as output. The regional normalised SUVR values and the MNI coordinates were then used as input to the Python application programming interface of the Nilearn [72] software. Using the connectome visualization capabilities of Nilearn we rendered this information using a glass brain view with the highest 30% of values shown; see Fig 14. A comparison with Fig 13 (bottom row) shows that characteristic PET features associated with Alzheimer’s disease [68, 69] are once more prominent in the connectome view of the top 30% of SUVR intensities.

Demonstrating that the mathematical model of (8)–(11) is capable of achieving distributions of toxic A*β* and *τ*P that resemble the PET data of AD patients is a multi-step process. We note that the demonstration endeavored here is illustrative and does not constitute a full validation of the model; it will, however, fully justify that the fitting of real-world data is within the capacity of the model. First, we set all regions in the connectome to a state of secondary tauopathy with the general synthetic parameters given by those in Table 3. The A*β*–*τ*P interaction parameter, *b*_3_, was modified in several regions. All of the modifications to *b*_3_ were symmetric; that is, they were made in both the left and right hemispheres of the corresponding region. The modified interaction parameters for connectome vertices in select secondary tauopathy regions are shown in Table 4. Finally, the connectome vertices in a total of five brain regions, in both hemispheres, were put into a state of primary tauopathy by changing the values of *b*_2_ and *b*_3_ to correspond to states in this regime. The primary tauopathy regions, and their parameters, are listed in Table 5.

**Table 3:** Comparison with ADNI Alzheimer’s patient PET data. General Synthetic parameters.

Parameter	Value	Parameter	Value	Parameter	Value	Parameter	Value
Healthy amyloid-*β* population parameters							
*ρ*	1.38	*a*_0_	1.035	*a*_1_	1.38	*a*_2_	1.38
Toxic amyloid-*β* population parameters							
*ρ*	0.138	${\tilde{a}}_{1}$	0.828	*a*_2_	1.38		
Healthy *τ*P population parameters							
*ρ*	1.38	*b*_0_	0.69	*b*_1_	1.38	*b*_2_	1.035
*b*_3_	4.14						
Toxic *τ*P population parameters							
*ρ*	0.014	${\tilde{b}}_{1}$	0.552	*b*_2_	1.035	*b*_3_	4.14

**Table 4:** Regional interaction parameter variation in secondary tauopathy.

Brain region ID and modified *b*_3_ value			
Pars Opercularis	7.452	Rostral middle frontal gyrus	6.707
Superior frontal gyrus	7.452	Caudal middle frontal gyrus	7.452
Precentral gyrus	5.589	Postcentral gyrus	3.726
Lateral orbitofrontal cortex	6.486	Medial orbitofrontal cortex	6.486
Pars triangularis	5.520e-6	Rostral anterior cingulate	6.210e-6
Posterior cingulate cortex	3.45	Inferior temporal cortex	13.11
Middle temporal gyrus	11.04	Superior temporal sulcus	8.97
Superior temporal gyrus	8.28	Superior parietal lobule	12.42
Cuneus	13.8	Pericalcarine cortex	13.8
Inferior parietal lobule	11.73	Lateral occipital sulcus	15.18
Lingual gyrus	13.8	Fusiform gyrus	7.59
Parahippocampal gyrus	11.04	Temporal pole	1.104e-5

**Table 5:** Primary tauopathy regions and parameters.

Brain region	*b*_2_	*b*_3_	Brain region	*b*_2_	*b*_3_
Entorhinal cortex	3.125	1.104e-5	Putamen	3.795	3.795
Pallidum	2.76	2.76	Precuneus	3.105	3.105
Locus coeruleus	1.38	1.38			

**Fig 15:**
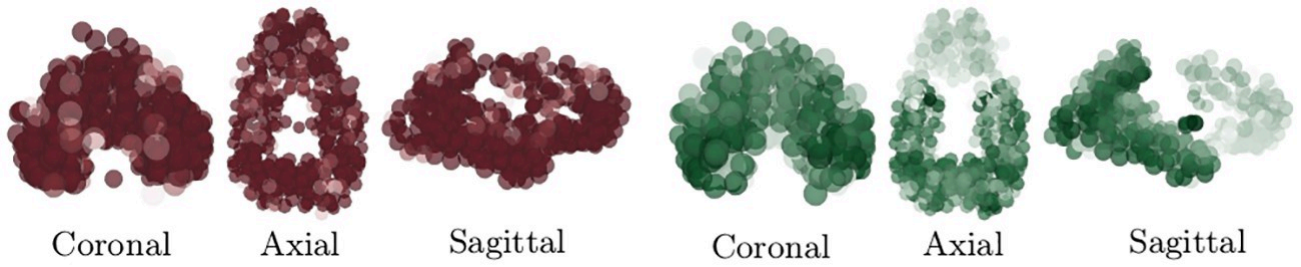
Results of a mixed-modality simulation. (left) Toxic A*β* population and (right) toxic *τ*P population are shown at time *t* = 78. The top 30% of nodal values are visible; darker colors correspond to higher values.

The connectome vertex parameters given by Table 3 and regional vertex parameter modifications pursuant to Tables 4 and 5 describe a mixed-modality mathematical model; the connectome graph contains vertices in a state of primary tauopathy and vertices in a state of secondary tauopathy. The model Eqs (8)–(11) were solved with the regional parameters, and modifications, described above. Seeding patterns for both A*β* and *τ*P are identical to those discussed at the beginning of the Alzheimer’s application section. The patient SUVR data, visualised on the conectome, is shown in Fig 14; the results of the simulation are shown in Fig 15 at time *t* = 78 in accordance with the mean age of the A*β* and *τ*P cross-sectional study parameters (c.f. Table 2); the highest 30% of values are visible. A comparison of Fig 13 (bottom row) and Fig 14 to that of Fig 15 shows that the model can indeed capture salient characteristics of Alzheimer’s disease proteopathy as indicated by SUVR intensity.

Thus, this preliminary result clearly demonstrates that the model can recover primary features of Alzheimer’s disease proteopathy and that more mathematically comprehensive analyses are warranted; for instance, investigations using (variational) Bayesian methods [75, 76] may be compelling for further study of the model alongside patient data for cognitively normal, mildly cognitively impaired, early and late onset Alzheimer’s disease cohorts.

### A simple model of local and non-local neuronal damage

This section briefly examines the use a simple measure of neuronal damage with the minimal level of complexity necessary to take into account both local and non-local effects. The intent is to explore the qualitative differences between the primary and secondary tauopathy regimes and the effect of varying: the toxification rate of A*β* on *τ*P; and the rate of aggregation due to non-local influence. The continuous Eq (3) were augmented (Methods, A continuous mathematical model) with a coarse-grained damage model (4). We recall that *q*(**x**, *t*) represents a first-order assessment for neuronal cell body damage vis-a-vis a, potentially variegated, set of coupled mechanisms. These mechanisms are not individually differentiated; however, they are assumed to be correlated with the presence of toxic A*β*, toxic *τ*P or with those mechanisms requiring both (c.f. the discussion surrounding Eq (4)).

The damage model (4) has several coefficients: *k*_1_ and *k*_2_ mediate the damaging effect of toxic A*β* and *τ*P respectively. The rate coefficient *k*_3_ reflects damage, such as the rate of neuronal death following over-excitation, resulting from the combined presence of toxic A*β* and toxic *τ*P. Finally, *k*_4_ determines the rate of transneuronal damage propagation; thus reflecting aggregate neuronal death as a result of communication disruption to and from regional neighbors.

In this illustrative example we consider the parameters

$$\begin{array}{c} \begin{array}{cccc} k_{1}=1\times 10^{-4}, & k_{2}=1\times 10^{-2}, & k_{3}=1\times 10^{-1}, & k_{4}=1\times 10^{-3}, \end{array} \end{array}$$ (2)

as a baseline from which to begin investigation. These parameters have been chosen to reflect a few clinical observations. First, *k*_1_ is chosen as significantly less than *k*_2_ to reflect the correlation [9, 10, 12, 13] of toxic *τ*P neurofibrillary tangles with various forms of neuronal damage (e.g. intracellular NFT-induced neuron death, atrophy etc). Second, toxic effects of *τ*P are increased in the presence of toxic A*β* [12, 26, 28, 29, 30, 31, 32, 33] thus, *k*_3_ is taken larger than *k*_2_.

**Fig 16:**
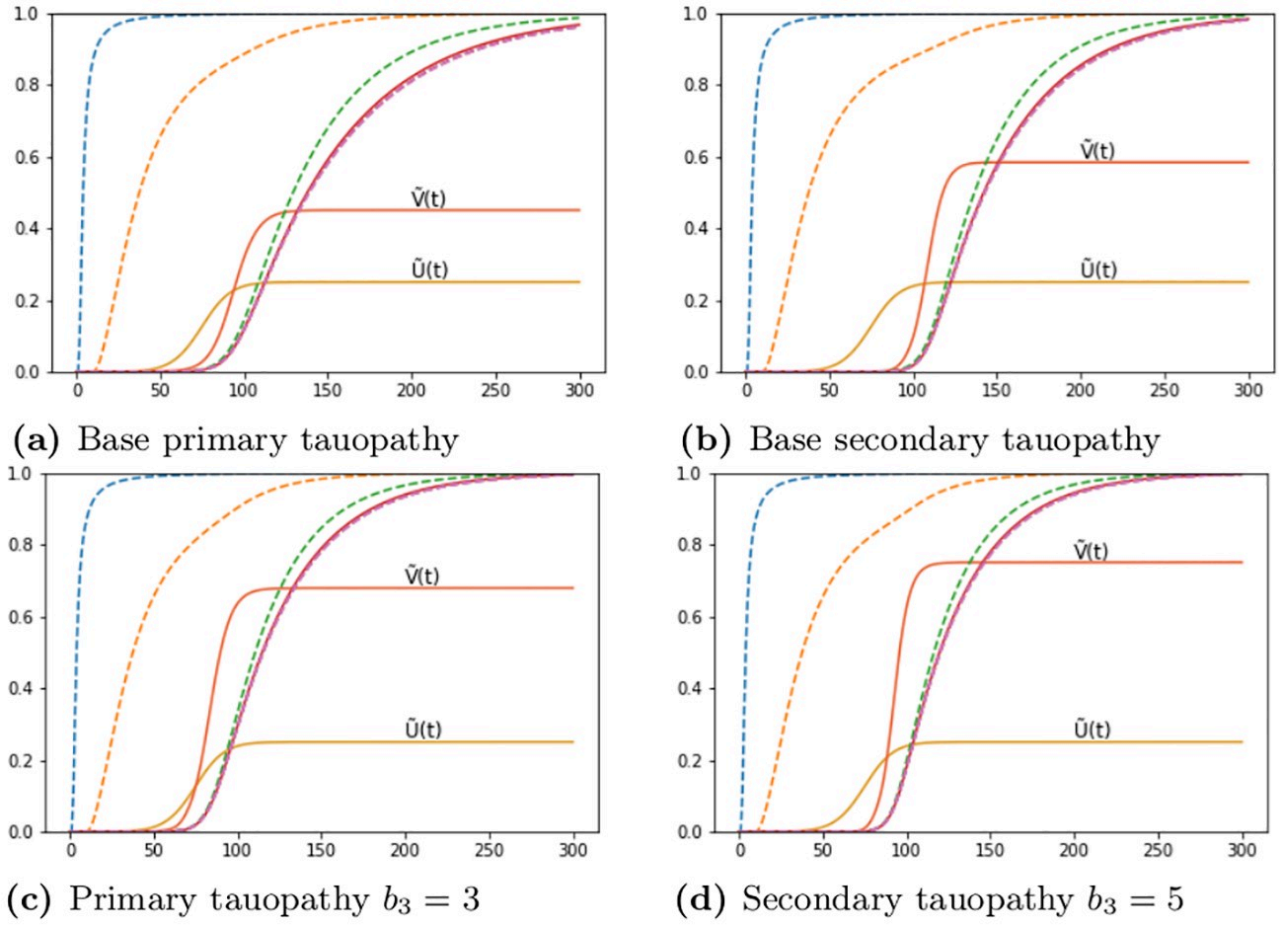
Aggregate damage in primary and secondary tauopathy. Aggregate damage (dashed; except *k*_4_ = 1 × 10^−3^ solid, red) curves in the base primary (a) and secondary (b) tauopathy patients. Damage with increase toxic protein interaction, *b*_3_, in primary (c) and secondary (d) tauopathy.

As a first point of enquiry: we consider our baseline tauopathy patient parameters (Table 1) and vary the deafferentation parameter *k*_4_ across three orders of magnitude from the initial value given in (2). Fig 16a and 16b show the results. Note that, in each subfigure, the dashed lines correspond, from left to right, to monotonically decreasing values of *k*_4_; the far left dashed curve is *k*_4_ = 1.0, the next curve to the right is *k*_4_ = 1 × 10^−1^, the next is *k*_4_ = 1 × 10^−2^, and so forth, down to the final (rightmost) curve corresponding to *k*_4_ = 1 × 10^−6^. In both figures the baseline deafferentation curve, *k*_4_ = 1 × 10^−3^, is instead solid (and red) for emphasis. Fig 16c and 16d show the effect of increasing *b*_3_; we have incremented *b*_3_ by two, from baseline, for each case. As expected an overall increase in toxic *τ*P, ${\tilde{v}}_{max}=0.679$ for primary tauopathy and ${\tilde{v}}_{max}=0.75$ for secondary, is observed with the increase in *b*_3_. However, the limiting behavior of the deafferentation baseline coefficient choice, *k*_4_ = 1 × 10^−3^, remains; which justifies our choice of *k*_4_ in (2).

**Fig 17:**
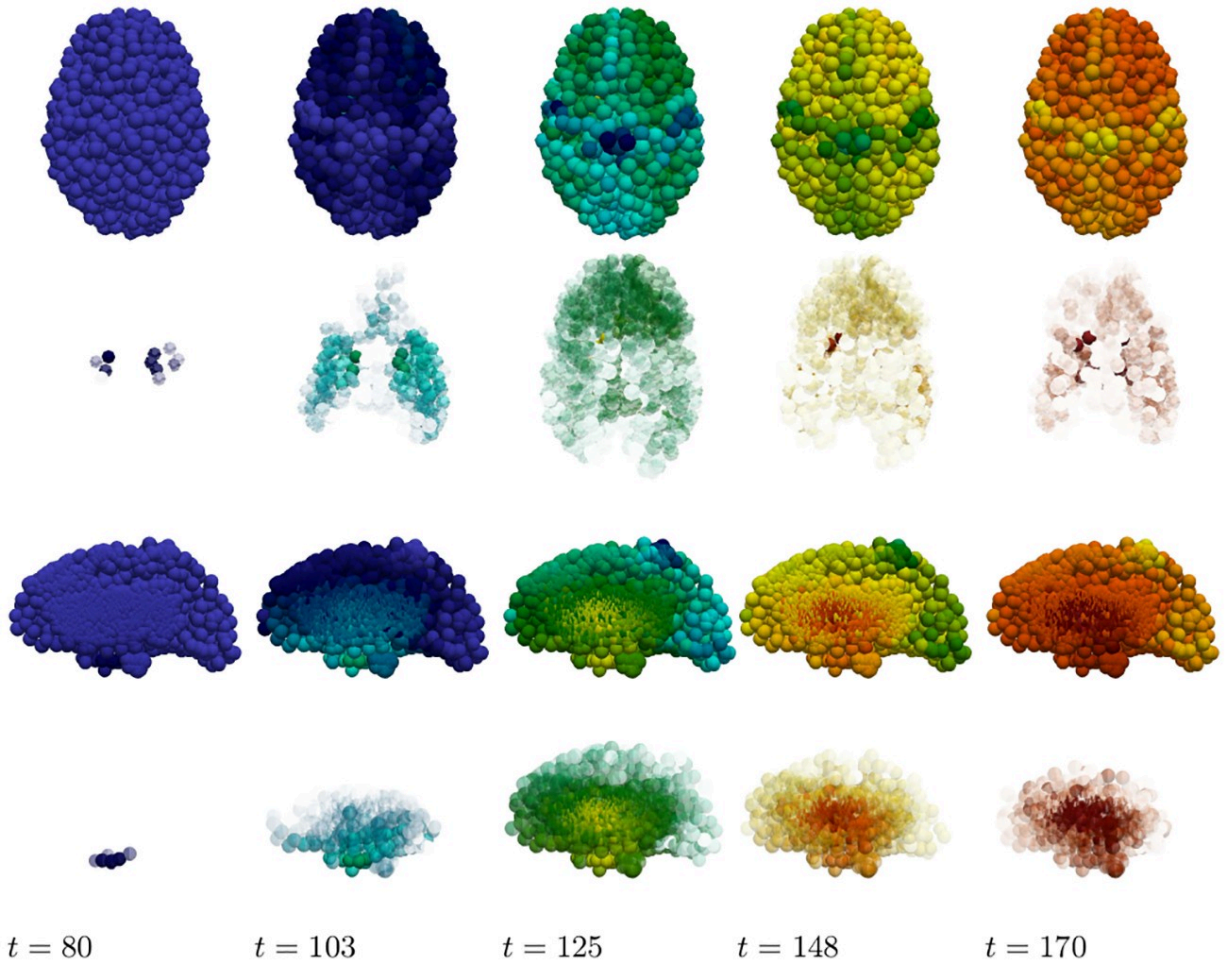
Damage progression in primary tauopathy. Horizontal plane view (top row) with opacity exaggerated (second row) progression. sagittal view (third row) with opacity exaggerated (fourth row) progression. Dark blue indicates the minimal damage value of *q* = 0.0; bright red indicates the maximum of *q* = 1.0. Intermediate values are: purple (*q* = 0.14), sky blue (*q* = 0.29), green (*q* = 0.43), yellow (*q* = 0.57), orange (*q* = 0.71), and dark red (*q* = 0.86).

**Fig 18:**
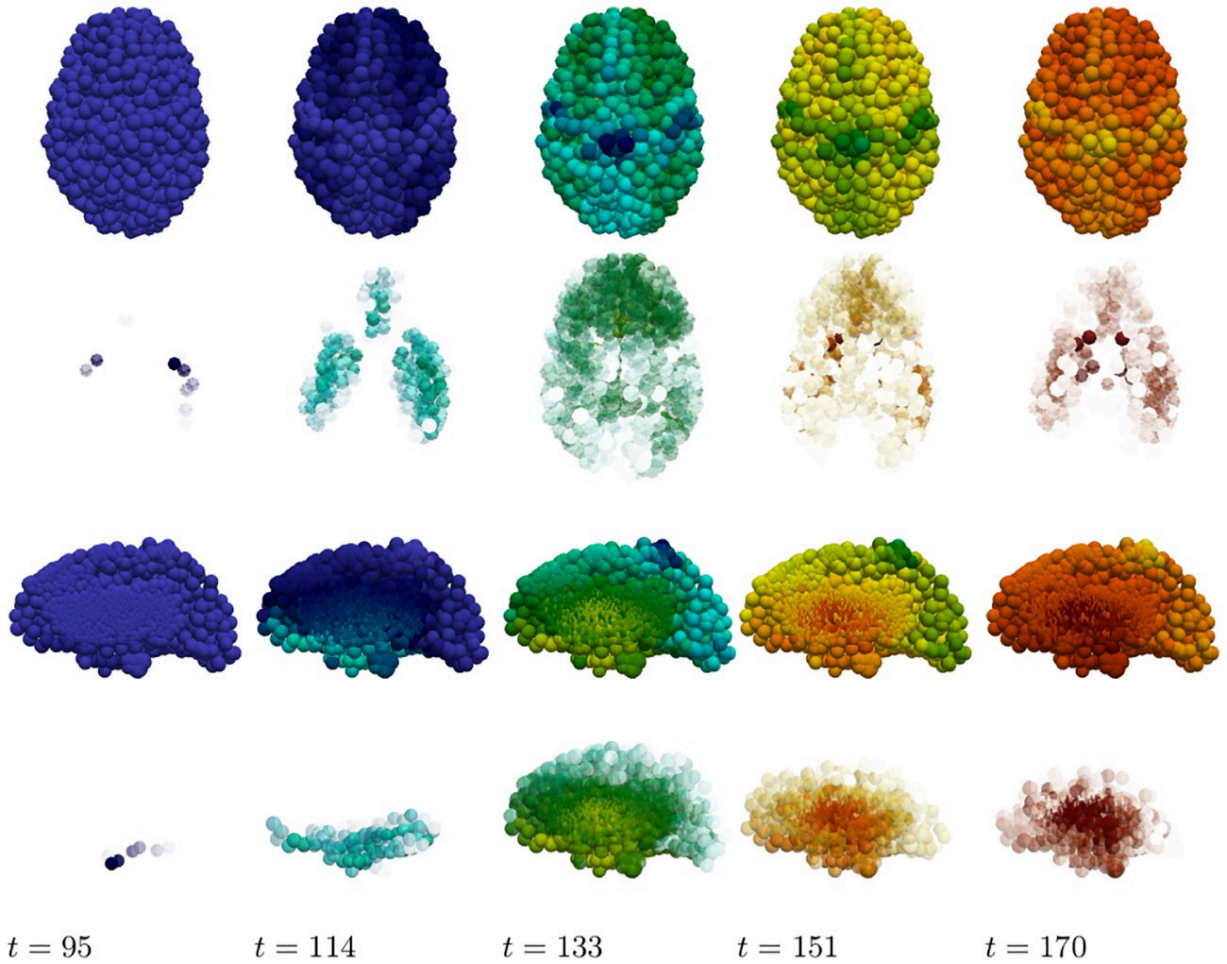
Damage progression in secondary tauopathy. Horizontal plane view (top row) with opacity exaggerated (second row) progression. sagittal view (third row) with opacity exaggerated (fourth row) progression. The color scale is identical to that of Fig 17.

The staging of the damage is presented in two figures: primary tauopathy in Fig 17 and secondary tauopathy in Fig 18. Each set of figures includes an overhead horizontal plane view in addition to a sagittal view of the right hemisphere. A visualization starting time was selected to coincide with the first visibility of 5% damage, in any nodes, while an ending time was selected such that the damage progression appeared qualitatively equal. Progression times are uniformly spaced within this interval to allow for a direct comparison between the damage distribution within the two regimes. An immediate observation is that a 5% damage detection is latent within the secondary model, starting at *t* = 95, compared to the primary tauopathy paradigm at *t* = 80.

It is challenging to discern differences between the fully opaque horizontal views of Fig 17 v.s. Fig 18; some discrepancies are apparent in the sagittal views, however. Relative opacity exaggeration is used to gain further insight. At each time the minimum and maximum damage, denoted *D*_min_ and *D*_max_, was computed across all regional nodes of the brain connectome; opacity was then set to linearly increase from: fully transparent at the average $\frac{1}{2}\left(D_{\text{min}}+D_{\text{max}}\right)$; to fully opaque at the maximum value *D*_max_. The resulting opacity exaggeration scheme shows, at each time step, the relative distribution of the most damaged regions.

The aforementioned opacity scheme leads to a further observations. First, the distribution of relative significant damage in primary tauopathy (Fig 17, second and fourth rows) is clustered more centrally to the toxic *τ*P seeding site of the transentorhinal cortex. Conversely, the distribution of relative significant damage in secondary tauopathy (Fig 18, second and fourth rows) is distributed in the direction of the temporobasal region; a site associated with A*β* seeding. As the disease progresses, *t* = 103 and *t* = 114 in Figs 17 and 18 respectively, we see two distinct differences: relative damage is more connected, in the horizontal plane, in addition to more diffuse in the coronal direction, of the sagittal plane, for the case of primary tauopathy; in secondary tauopathy the relative damage in the horizontal plane forms three distinct clusters while severe damage in the sagittal plane is follows the temporobasal and frontomedial directions.

It is increasingly difficult to visually detect qualitative patterns in later stages of significant damage progression; that is, *t* ≥ 125 for primary tauopathy and *t* ≥ 133 for secondary. Nevertheless it appears that late stages, *t* = 148 and *t* = 170, for primary tauopathy display a more diffuse distribution of significant relative damage away from the transentorhinal region; whereas late secondary tauopathy, *t* = 151 and *t* = 170, show more comparative significant damage in the areas associated with A*β* initial seeding.

Taken collectively: these observations suggest that damage onset and the relative distribution of severe damage may offer distinct points of view for application modelling to both typical Alzheimer’s disease along with its neuropathological subtypes [77, 78].

## Discussion

In this section we reflect on the analytic and computational results of the manuscript. We first list some advantages and limitations of the current perspective; in we then discuss several results in the context of the current literature and offer further questions and brief concluding remarks. The proposed model is based on physical protein aggregation kinetics; the simplest such two-famiy-two-species interacting protein model one could posit. Nevertheless, the model is mathematically sophisticated enough to evince two distinct pathology regimes, termed primary and secondary tauopathy, of potential clinical interest. After discretizing (3) on a structural connectome: an approachable system of non-linear ordinary differential Eqs (8)–(11), emerges which can be solved using standard mathematical software; such as Mathematica or Matlab. As a result, we expect the model to be widely appealing to the computational neurodegenerative disease community as a starting point for gaining further insight into protein-protein interactions in the context of Alzheimer’s disease.

### Advantages and limitations

The deterministic nature of the model, c.f. (3), has at least three distinct advantages over complex, stochastic models: first, the reaction terms of (3) represent simplified [49, 50], but physical, protein aggregation kinetics with a basis in experimental measurement [50, 52, 53, 54, 56]; second, the connectome-discretized Eqs (8)–(11), can also be readily implemented using off-the-shelf mathematical software (e.g. Mathematica or Matlab, etc). Thus, (8)–(11) are easily approachable and do not require probabilistic postulations, based on data or otherwise, regarding underlying distributions. A third advantage is that (8)–(11) are amenable to an a-priori mathematical analysis. This analysis is immutable in nature and much can be observed as a result of using standard methods from the theory of ordinary differential equations and non-linear diffusion-reaction systems. Conversely, probabilistic models may need extensive tuning, reformulation or data curation in order to determine a model’s emergent properties. An independent investigation, i.e. model fitting and application, founded on datasets with differing fidelity may produce divergent results. Such models essentially act in service to deeply mine a set of data but are not always directly helpful to elucidate the impact of individual disease mechanisms.

Conversely, (8)–(11) has inherent limitations. As discussed in recent literature: [35, 79] there are challenges surrounding the acquisition of the parameters in deterministic models such as (8)–(11). In vitro kinetic parameters, regulating the multiplication and growth of several proteins, have been ascertained for A*β*, *τ*P, *α*-synuclein and others; c.f. the citations in [79]. If we disregard the clearance terms in the prototypical heterodimer model, (8) and (9), then precisely two kinetic coefficients remain: source production (*a*_0_) and healthy-to-toxic conversion (*a*_2_). The in-vitro experimental estimation of protein-specific aggregation kinetic parameters, however, typically relies on more complex theoretical models: consisting of at least five kinetic parameters; and an infinite number of equations (c.f. for instance [48, Sec. 3.2]). It is therefore not immediately clear how to obtain explicit values for the kinetic rate parameters of (8)–(11). Asymptotic expansions have provided links between the rate coefficients of other more complex models and their simpler counterparts, c.f. [Sec. 2.2] [47], and such approaches may provide insight into reducing experimental parameters from the five-parameter models [79, 48] to those of (8)–(11). A further complication, though, is that even if explicit, experimentally verified, in-vitro parameters were available for (8)–(11) these do not necessarily translate into the correct parameters in vivo [79] where indirect, and locally varying, mechanisms (such as aggregates interacting with cells, or the effects of inflammation on aggregation dynamics) may play a role in altering the associated rates; either globally or regionally.

### Observations and open questions

Neurodegenerative diseases are complex and multi-scale processes. The point of view of (8)–(11) is to reduce this complexity by considering a collection of aggregate mechanisms and their implications. For instance (3) can be viewed, more conversationally, as the following collection of general mechanisms: (a) there exists two protein families; (b) each family has a healthy and toxic species; (c) these species are produced and cleared at some aggregate (regional) rate (d) any movement of these species, within the brain, is primarily determined by the macroscale axonal structure; (d) healthy proteins within a family can become toxic, at some (regional) rate, based on the presence of other toxic proteins of that family; and (e) the conversion of healthy-to-toxic proteins, for the second family, is further influenced by the presence of the toxic population of the first family. The current literature suggests that this collection of observations outlines a minimal prion-like model of Alzheimer’s disease progression.

In section the discussion on advantages and limitations it was mentioned that one advantage of simple deterministic models, such as (8)–(11), is that the impact of individual mechanisms can be elucidated and several emergent behaviors can be ascertained a priori. Models such as (3) therefore lead, naturally, to additional questions and serve as a trailhead for further development. The first, and critical, observation is that: (8)–(11) implies that the local balance of clearance, e.g. (23), plays a fundamental role in disease initiation. In light of the seminal work of Braak and Braak [11] this leads naturally to the question: what are the local (toxic *τ*P) clearance properties characterizing the transentorhinal region, (which defines the early Braak stages) and how do these local properties differ from other regions? Aspects of the fine-scale clearance mechanisms of toxic *τ*P remain unclear or are even controversial [80, 81, 82, 83]. Nevertheless, our simple framework reinforces the sentiment echoed by experimentalists: that understanding these processes may be critical to a mechanistic understanding of the initiation of the disease cascade.

A second observation emerging from (8)–(11) is that the progression of Alzheimer’s disease may consist of a confluence of brain regions simultaneously in differing states characterized by contrasting fundamental dynamics. In particular (Methods, Stability and Disease phenomenology) even our simple model of AD development suggests potentially complex disease phenomenology; one where *τ*P can evolve independently of A*β* (termed primary tauopathy) and one where *τ*P depends intrinsically on the presence of A*β*. Furthermore, the line between these two regimes is demarcated by: the balance of local clearance; and the degree of local influence of A*β* on the toxification of *τ*P [12, 26, 30, 31, 32, 33] as expressed by the bulk parameter *b*_3_. Depending on these local attributes we could have some areas of the brain in a state of primary tauopathy and others in a state of secondary tauopathy; the latter regions having their tauopathy delayed until a toxic A*β* population is established while the former regions are free to develop toxic *τ*P and NFT independently. This leads naturally to another fundamental line of further enquiry: what are the simplest additional relations, extended (8)–(11), needed to suitably describe the evolution of clearance and toxicity rates alongside protein pathology?

Our simple mathematical model suggests, as a third observation, that the rate of toxic A*β*-*τ*P interaction (i.e. *b*_3_) is not a passive facet of disease phenomenology but, rather, may play a much more integral role. We have already discussed, above, that *b*_3_ plays a role in secondary tauopathy; it can do this by lowering *v*_4_, in (17), thus ensuring that ${\tilde{v}}_{4}$ is an admissible state. Other interesting observations regarding *b*_3_ were discussed in the results section (c.f. A simplified model of Alzheimer’s disease proteopathy). The observation regarding the impact of *b*_3_ in local and non-local neuronal damage formation is straightforward so we will mention it first; namely, that transneuronal damage propagation in the model has a ‘minimum speed’ in both primary and secondary tauopathy; and that increasing *b*_3_ has no effect. In particular Fig 16, and the surrounding discussion, suggests that lowering the ‘transmission coefficient’, reflected in *k*_4_, below a certain threshold does not lower transneuronal damage; and that increasing *b*_3_, for a fixed choice of *k*_4_, does not increase the overall propagation of damage. These details lead to a somewhat interesting observation: that the rate of neuronal damage from the structural network topology of the brain may exhibit a baseline, or minimum, value; independent of the details driving local damage (e.g. from local toxicity).

We also observed two features of tauopathy directly related to *b*_3_; the time of onset and the *τ*P ‘invasion window’. We recall that the time of onset is defined as the first appearance of toxic *τ*P while the ‘invasion window’ is the timespan starting at a 1% toxic *τ*P concentration and terminating when the asymptotic steady-state value is achieved. In primary tauopathy, disease onset time is virtually unaffected by varying *b*_3_ whereas increasing levels of *b*_3_ shortens the tauopathy invasion window. In addition, the asymptotic concentration value of toxic *τ*P increases with *b*_3_ so that, overall, increasing *b*_3_ implies that a more severe tauopathy will develop, faster, at a similar starting point in time (c.f. Fig 8). The picture in secondary tauopathy is different. We see, again, that increasing *b*_3_ does increase the severity of the tauopathy (Fig 10b); however, this is where the similarities with primary tauopathy end. First, as *b*_3_ increases the time of onset decreases (Fig 10b). Second, the invasion window in secondary tauopathy does not decrease monotonically with decreasing *b*_3_ (Fig 10c); rather, we see the invasion window start time and end both decrease, with increasing *b*_3_, while the start time decay and end time decay, relative to increasing *b*_3_, is different (Fig 11). This is the cause of the initial drop, from *b*_3_ = 0.75 to *b*_3_ = 3.0, of the invasion window in Fig 10c followed by an increase to a steady invasion window length circa *b*_3_ = 12. The observation that increased *b*_3_ can decrease the time of onset in secondary tauopathy, which requires the presence of A*β*, while also impacting the invasion window time is reminiscent of the effects associated to the presence of particular Apoliprotein E (APOE) allele configurations. For instance: APOE *ϵ*4 carriers are more likely to develop AD; toxic A*β* production and deposition is more abundant in APOE *ϵ*4 carriers; and APOE *ϵ*4 exacerbates A*β*-related neurotoxicity [84].

In the results subsection ‘a mixed model comparison to Alzheimer’s diseased patient data’ we discussed a mixed-modality instantiation of the model (8)–(11), with some regions in a state of primary tauopathy with all others in a state of secondary tauopathy, using hand-selected synthetic parameters. It was demonstrated that such a mixture of states can reproduce salient features seen in Alzheimer’s disease; in particular, the distinct distribution patterns [68, 69] of 18F-AV-1451 radiotracer are clearly observed. This observation suggests that the model of (8)–(11) is sufficiently rich and implies that the undertaking of a comprehensive data fitting and comparison study is both well warranted and an optimistic endeavor. It is interesting to note, though, that the distinction between primary and secondary tauopathy is not simply one of differently-valued parameters; in particular the two states are differentiated by the balance of clearance inequalities (Methods, Stability). In particular, we have ${\tilde{a}}_{1}/a_{2}<a_{0}/a_{1}$ and ${\tilde{b}}_{1}/b_{2}<b_{0}/b_{1}$ for primary tauopathy; for secondary tauopathy the latter inequality changes sign to $b_{0}/b_{1}<{\tilde{b}}_{1}/b_{2}$. This observation implies that an arbitrary parameter fitting could produce accurate results, compared to data, while still being questionable since the fitting would imply secondary characteristics regarding regional clearance attributes which may or may not hold. It would therefore be beneficial to carefully consider a data-based measure of regional clearance, for both A*β* and *τ*P, when selecting a data fitting method; possibly incorporated as a constraint or as part of a cost functional. Nevertheless, the dual regimes of primary and secondary tauopathy provide a verdant backdrop for further modelling endeavors; both in terms of fitting to clinical imaging data and to probing and modelling possible ties between *b*_3_, APOE configuration, and secondary tauopathy.

In the introduction section we mentioned that the role of Tau in AD formation, and development, is beginning to be recognized as a potentially significant factor. Despite this, open questions about the nature of tau, and tauopathy, in AD remain. For instance: one could argue that healthy *τ*P, being bound to microtubules, should not be diffusing at all. The literature suggests that even healthy tau in healthy neurons exhibit mobility within the cell [85], is secreted into the extracellular space [86, 87], and that extracellular tau is taken up by neighboring neurons [88]; even in the absence of pathology. It is not entirely clear what the correct choice of *ρ*, in (7), should then be. Despite the literature seeming to suggest that *ρ* > 0 one could still insist that *ρ* ≪ 1, or possibly even assert that *ρ* = 0, should be chosen in (7) for the graph Laplacian of (10). Regarding impacts to the model: this perspective alone would not affect any of the analytic observations (Methods, An Analysis of the continuous model). Indeed, if *v* is the vector whose *j*^th^ entry is the healthy tau concentration, *v*_*j*_, in node *j* then if *v* is constant, or nearly so, then the graph Laplacian applied to *v* is zero, or nearly so, regardless of the value of *ρ*. Thus, since all of the nodes in the computational investigations discussed in the results section had their healthy tau populations set to the same constant value: the effect of any healthy tau diffusion in the simulation results there would be expected to be entirely negligible as well.

The nature of the rates for healthy tau production and clearance, in the literature, are also not fully understood. Indeed, the visual confirmation of the mRNA machinery for localized transcription [89] of tau in axons, and growth cones, is less than two decades old; clarifying important aspects of tau clearance, both healthy and diseased, is an ongoing process [80, 82, 83]. Our results indicate that violation of the balance of clearance inequalities, (23), is fundamental for disease initiation and phenomenology; for instance: if healthy tau were not regenerated, so that *b*_0_ = 0, then the regime of ‘primary tauopathy’ (which requires that ${\tilde{b}}_{1}/b_{2}<b_{0}/b_{1}$) would be an impossibility. This would imply that, in the context of our model, that the development of all tauopathies would require an accompanying amyloidopathy and would seem to preclude those tauopathies which are mostly dominated by toxic *τ*P spreading [8]. It has been observed that: tau is expelled from neurons [86], including healthy ones, on a periodic basis [87]; and that tau plays a role in cell signalling, cell polarity, synaptic plasticity and the regulation of genomic stability [90, 91]. These observations, alongside recent work in adult neurogenesis [92], give good reason to suspect that both *b*_0_ > 0 and *b*_1_ > 0; at least in the healthy brain and early in disease progression. An open question, though, is how these quantities may change with disease progression. For instance, one could extend the current model by coupling *b*_0_ with the damage coefficient *q*; reflecting the fact decreased healthy tau synthesis could result from neuronal loss, and the decline of neurogenesis [92], throughout AD progression.

**Fig 19:**
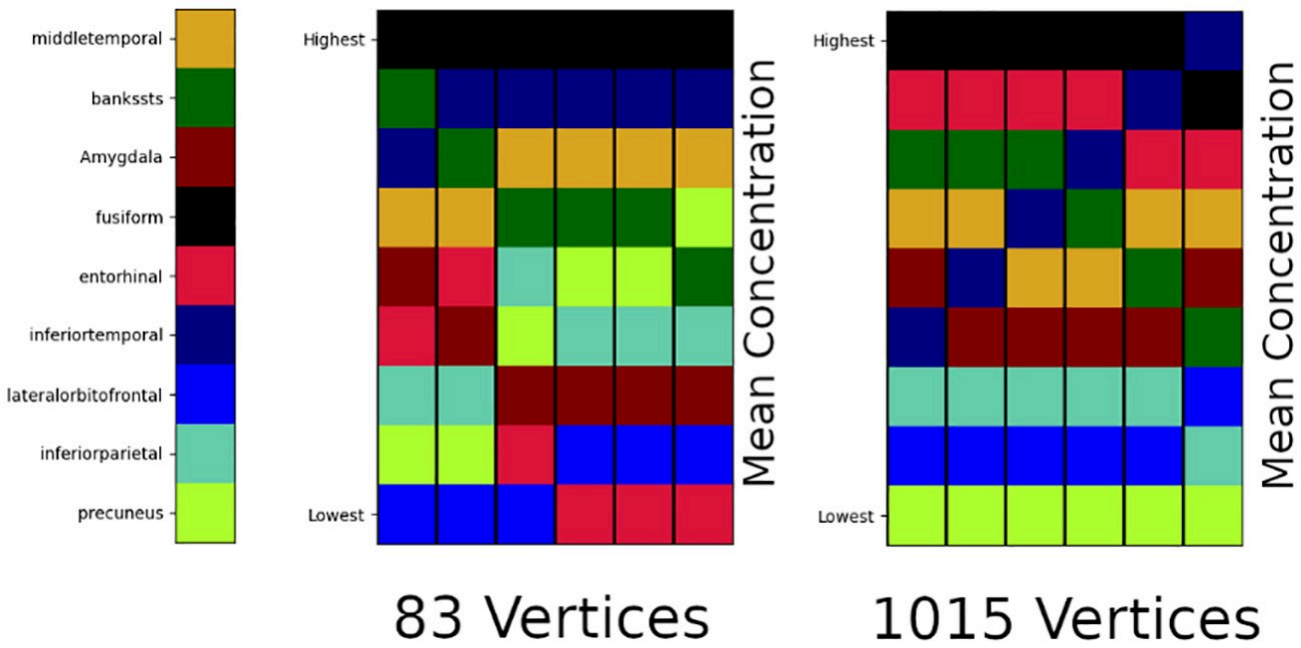
Toxic *τ*P average regional concentration; six fixed time points. 83 (left) versus 1015 (right) vertex connectomes.

The final observation we mention regards an open question surrounding the imaging, and construction, of the structural connectomes used in such network models. We have used an often cited connectome [58, 59]. This connectome is available in various resolutions; the lowest of which consists of 83 vertices (regions of interest) while the highest resolution case, which we have used here, consists of 1015 vertices. However, there are apparent differences in both A*β* and *τ*P staging when solving equations, such as (8)–(11), on the low versus high resolution connectomes. We used the simple, illustrative parameters described for primary tauopathy (Results, A simplified model of Alzheimer’s disease proteopathy) and recorded the average regional tau concentration at six fixed time points; the time points were selected to span disease progression. Fig 19 shows the results of this tau staging experiment for nine regions.

In Fig 19, we can see that the two different resolutions of connectomes, derived from the same set of patient data, offer distinct staging patterns for tau progression. This implies that the connectome itself may play a significant role in retrieving results that match clinical data. Since the parameters of (8)–(12) have a physiological interpretation a simple ‘fitting’ to available clinical data is not satisfactory. Fig 19 suggests that developing a more rigorous understanding of computational staging behavior should be endeavored seriously and from first principles. Validating computational (tau) staging behavior, at different connectome resolutions, against clinical standardized-uptake-value-ratio (SUVR) studies, e.g. [93], is an important next step.

### Concluding remarks

We have presented a novel, minimal, and deterministic theoretical mathematical model of protein propagation that includes two interacting protein species. The model is motivated by recent experimental evidence regarding the potential importance that interactions between A*β* and *τ*P may play in the development of AD pathology [14, 16, 23, 24, 25]. The primary contributions of the current manuscript are: clearly, and mathematically, establishing the intrinsic dependence of the model on the balance of clearance inequality, (23), and the stability analysis of the modes of primary and secondary tauopathy (Methods, Stability and Disease Phenomenology); and establishing the speed of propagation of toxic fronts (Methods, Front propagation). Further novel contributions of interest include: demonstrating qualitative properties of disease propagation and damage, in primary and secondary tauopathy (Results, A simplified model of Alzheimer’s disease proteopathy), using globally constant, but non-physical, parameters; and demonstrating that the model can achieve tau distributions that reflect canonical patterns in Alzheimer’s patient data (Results, A mixed model comparison to Alzheimer’s diseased patient data). In particular, we have seen that the topology of the brain connectome leads to complex behavior in both pathological regimes. Finally, we have contextualized numerous analytic and computational observations with reference to the current literature and drawn attention to open avenues of further research suggested by the current work.

Alzheimer’s disease is a complex and multi-scale disease. The need for mathematical models, presenting observed disease characteristics, that are computationally tractable is pressing. Our findings suggest that further enquiry into both protein interaction and clearance processes is an important path forward in elucidating key mechanisms in the progression of these diseases. Due to the ease of implementation of (8)–(12), and the widespread interest in computational neurodegenerative disease, we hope that this model will be appealing, to the community, for probing the nuances of protein-protein interactions in neurodegenerative disease development.

## Methods

### A continuous mathematical model

**Fig 20:**
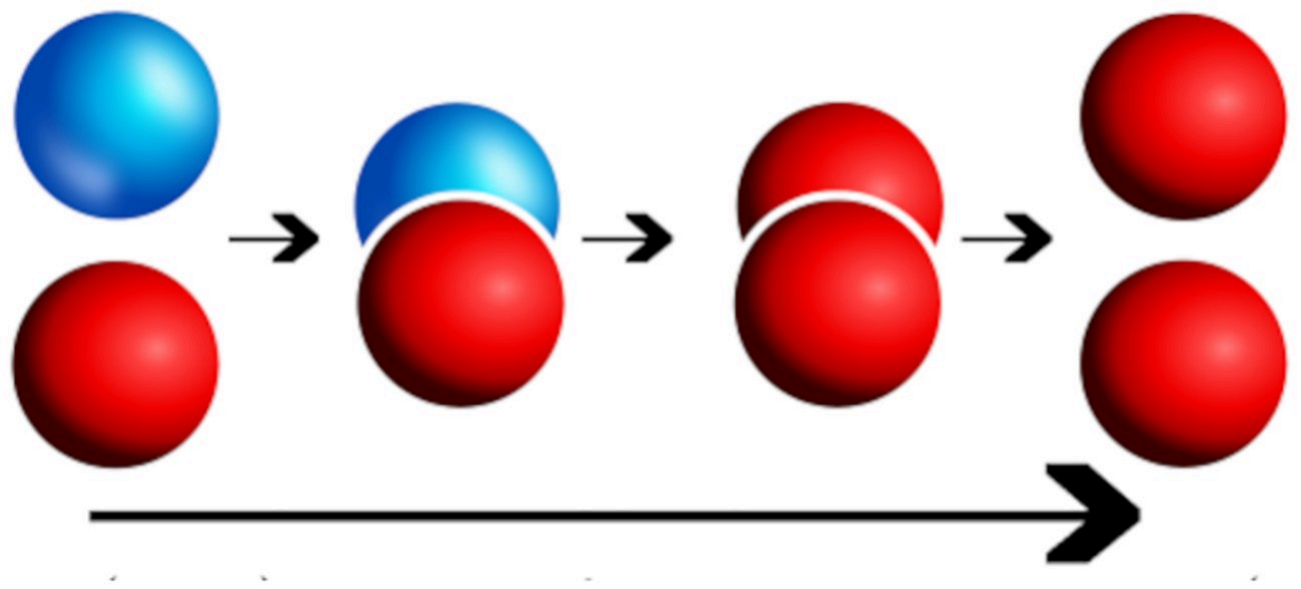
Kinetics of the heterodimer model. Healthy protein (blue) and misfolded toxic protein (red) transition to two toxic proteins (long arrow) via, from left to right, the kinetics of: recruitment, induced misfolding, and fragmentation.

The simplest possible deterministic aggregation model accounting for the interaction of two protein families, each consisting of a healthy and toxic population, is the heterodimer model [49, 51, 50]. In the heterodimer model: a toxic, misfolded seed protein recruits a healthy protein, induces misfolding, and then fragments; ultimately producing two copies of the misfolded toxic variant. The heterodimer model views these three processes as a single step and expresses this step as an overall mean rate of reaction; such rates can be determined experimentally. Indeed, measuring the mean rates of protein self-aggregation mechanisms, providing best-fit mean aggregation dynamics to deterministic models such as the heterodimer model, is a thriving field of contemporary research [50, 52, 53, 54, 55, 56] and our choice of a deterministic model is inspired by such work. Fig 20 demonstrates the primary molecular mechanism of the heterodimer model; the healthy (blue) protein is approached by the toxic (red) protein and undergoes three separate transitions (small arrows) which are treated as a single transition (long arrow) taking a healthy protein to a misfolded, toxic state.

We are interested in the interaction between two different protein families; motivated by the A*β* and *τ* interactions observed in AD. Towards this end we will consider two heterodimer models: one for A*β* and one for *τ*P. These two models will be coupled together by a single term reflecting that the formation of new toxic *τ*P can be enhanced by the presence of A*β*. The heterodimer model was originally posed [49, 50] as a continuous, non-linear, partial differential reaction-diffusion equation for a single protein. To define the model for our two protein families: let $\Omega \subset R^{3}$ be a spatial domain of interest and, for **x** ∈ Ω and time $t\in R^{+}$, we denote by *u* = *u*(**x**, *t*), and *v* = *v*(**x**, *t*) the concentration of healthy A*β* and *τ*P. Similarly, we denote by $\tilde{u}=\tilde{u}\left(x,t\right)$, and $\tilde{v}=\tilde{v}\left(x,t\right)$, the concentration of toxic A*β* and *τ*P, respectively. Then, the concentration evolution is governed by

$$\begin{array}{c} \begin{array}{ccccccc} \frac{\partial u}{\partial t} & = & \nabla \cdot (D_{1}\;\nabla u) & + & a_{0} & - & a_{1}\;u-a_{2}\;u\;\tilde{u},\\ \frac{\partial \tilde{u}}{\partial t} & = & \nabla \cdot ({\tilde{D}}_{1}\;\nabla \tilde{u}) & & & - & {\tilde{a}}_{1}\;\tilde{u}+a_{2}\;u\;\tilde{u},\\ \frac{\partial v}{\partial t} & = & \nabla \cdot (D_{2}\;\nabla v) & + & b_{0} & - & b_{1}\;v-b_{2}\;v\;\tilde{v}-b_{3}\;\tilde{u}\;v\;\tilde{v},\\ \frac{\partial \tilde{v}}{\partial t} & = & \nabla \cdot ({\tilde{D}}_{2}\;\nabla \tilde{v}) & & & - & {\tilde{b}}_{1}\;\tilde{v}+b_{2}\;v\;\tilde{v}+b_{3}\;\tilde{u}\;v\;\tilde{v}. \end{array} \end{array}$$ (3)

The first two equations, above, correspond to the usual heterodimer model for the healthy and toxic variants of the protein *u*; note that the second heterodimer model, for the variants of protein *v*, deviates from the form of the first by a single balanced term with coefficient *b*_3_. The system (3) could apply to any two families of interacting proteins; though the model is inspired by AD. The parameters are as follows: (*a*_0_, *b*_0_) are the mean production rates of healthy proteins, ($a_{1},b_{1},{\tilde{a}}_{1},{\tilde{b}}_{1}$) are the mean clearance rates of healthy and toxic proteins, and (*a*_2_, *b*_2_) reflect the mean conversion rates of healthy proteins to toxic proteins. The coupling between the two, otherwise separate, heterodimer models for A*β* and *τ*P, is realized via *b*_3_. The *b*_3_ predicated terms arise from the mode of interaction assumption, c.f. **M1** above, dictating that the presence of A*β* augments the conversion process of healthy *τ*P to toxic *τ*P. We note that toxic A*β* acts as an enzyme in this process and is therefore not depleted. In the absence of production and clearance maps, we assume that all these parameters are constant in space and time. The symmetric diffusion tensors **D**_1,2_ and ${\tilde{D}}_{1,2}$ characterize the spreading of each proteins. For isotropic diffusion, these tensors are a multiple of the identity, **D**_1,2_ = *d*_1,2_**1** and ∇ ⋅ (**D**_1,2_ ⋅ ∇(*u*)) = *d*_1,2_Δ(*u*) is the usual Laplacian operator (similarly for $\tilde{u}$, *v* and $\tilde{v}$) For anisotropic diffusion, the eigenvector with the largest eigenvalue describes the direction of faster diffusion which is used to model preferential propagation along axonal pathways [37].

The coupled system of Eq (3) dictates the spread, genesis, and clearance of two healthy species, *u* and *v*, and two toxic species, $\tilde{u}$ and $\tilde{v}$, of proteins throughout the domain Ω. The presence of toxic proteins near a point **x** ∈ Ω can disrupt the extracellular environment of neurons near **x** and impair their intracellular function. A broad range of coupled effects can contribute to neuronal impairment; including: chronic inflammation, erosion of the blood-brain barrier surrounding vessels, accelerating tau hyperphosphorylation, disrupting normal synaptic efficacy, and deafferentation, among others. A hallmark of neurodegenerative proteopathies is cognitive decline; propelled by the various coupled effects induced by the presence of toxic aggregates and the widespread erosion of neuronal integrity. The nuanced coupling between these disparate deleterious effects is not well understood; nevertheless, we employ the observation that such effects are generally correlated with larger concentrations of misfolded aggregates to define a gross measure of regional neuronal ‘damage’ denote by *q*(**x**, *t*) ∈ [0, 1]. This damage variable takes the perspective that *q*(**x**, *t*) = 0 signifies that the neurons in a neighborhood of **x** ∈ Ω are functional and healthy whereas *q*(**x**, *t*) = 1 implies that neurons near **x** have reached a fully-degenerate asymptotic state whereby they are either no longer functioning or fully deceased. For the evolution of the damage we assume a simple, first-order rate model:

$$\begin{array}{c} \dot{q}=(k_{1}\tilde{u}+k_{2}\tilde{v}+k_{3}\tilde{u}\;\tilde{v}+k_{4}A\left(q\right))\left(1-q\right),\;q\left(x,0\right)=0. \end{array}$$ (4)

When *k*_4_ = 0: the evolution Eq (4) can be seen as a first order reaction model, i.e. exponential decay, for the transformed variable $\hat{q}=\left(1-q\right)$; the associated rate of decay is then dependent on the deposition concentration of pattern of $\tilde{u}$ and $\tilde{v}$. The first two parameters in (4) denote contributions to neuronal dysfunction, near **x**, due to presence of isolated toxic aggregates; while the third term accounts for contributions requiring, or accelerated by, the presence of both toxic species aggregate species together. Thus, the third term engenders both toxic effects M2 and M3; c.f. the introduction section. The first three terms of (4) account for neuronal dysfunction in a neighborhood of the point **x** while the last term, A(q), incorporates non-local contributions, such as transneuronal degeneration, whereby the impairment, or death, of neighboring neurons can increase [57] the probability of impairment near **x**; thus leading to an increased mean rate of local decline. This nonlocal term does not have a simple representation within the continuous framework as the positions of neuronal bodies is not explicitly encoded. However, we will see that in the discrete case, there is a natural way to take this effect into account and we will delay the discussion of this term until the next section.

### A network mathematical model

A simple coarse-grain model of the continuous system can be obtained by building a network from brain data. The construction is obtained by defining nodes of the network to be regions of interest in the domain Ω, typically associated with well-known areas from a brain atlas. The edges of this network represent axonal bundles in white-matter tracts. The brain connectome is then modeled as a weighted graph G with *V* nodes and *E* edges obtained from diffusion tensor imaging and tractography. A network approximation of the diffusion terms, having the general form ∇ ⋅ (**D**∇*u*) or similar, in the system (3) will be constructed by means of a weighted graph Laplacian. The weights of the weighted adjacency matrix **W**, used to construct the graph Laplacian, are selected as the ratio of mean fiber number *n*_*ij*_ by mean length squared, $l_{ij}^{2}$, between node *i* and node *j*. That is:

$$\begin{array}{c} W_{ij}=\frac{n_{ij}}{l_{ij}^{2}},\;i,j=1,\ldots ,V. \end{array}$$ (5)

The choice of weights, above, are consistent with the inverse length-squared dependence incurred by canonical discretizations of the continuous Laplace (diffusion) operator appearing in (3). The weighted degree matrix is the diagonal matrix with elements

$$\begin{array}{c} D_{ii}=\sum_{j=1}^{V}W_{ij},\;i,j=1,\ldots ,V. \end{array}$$ (6)

**Fig 21:**
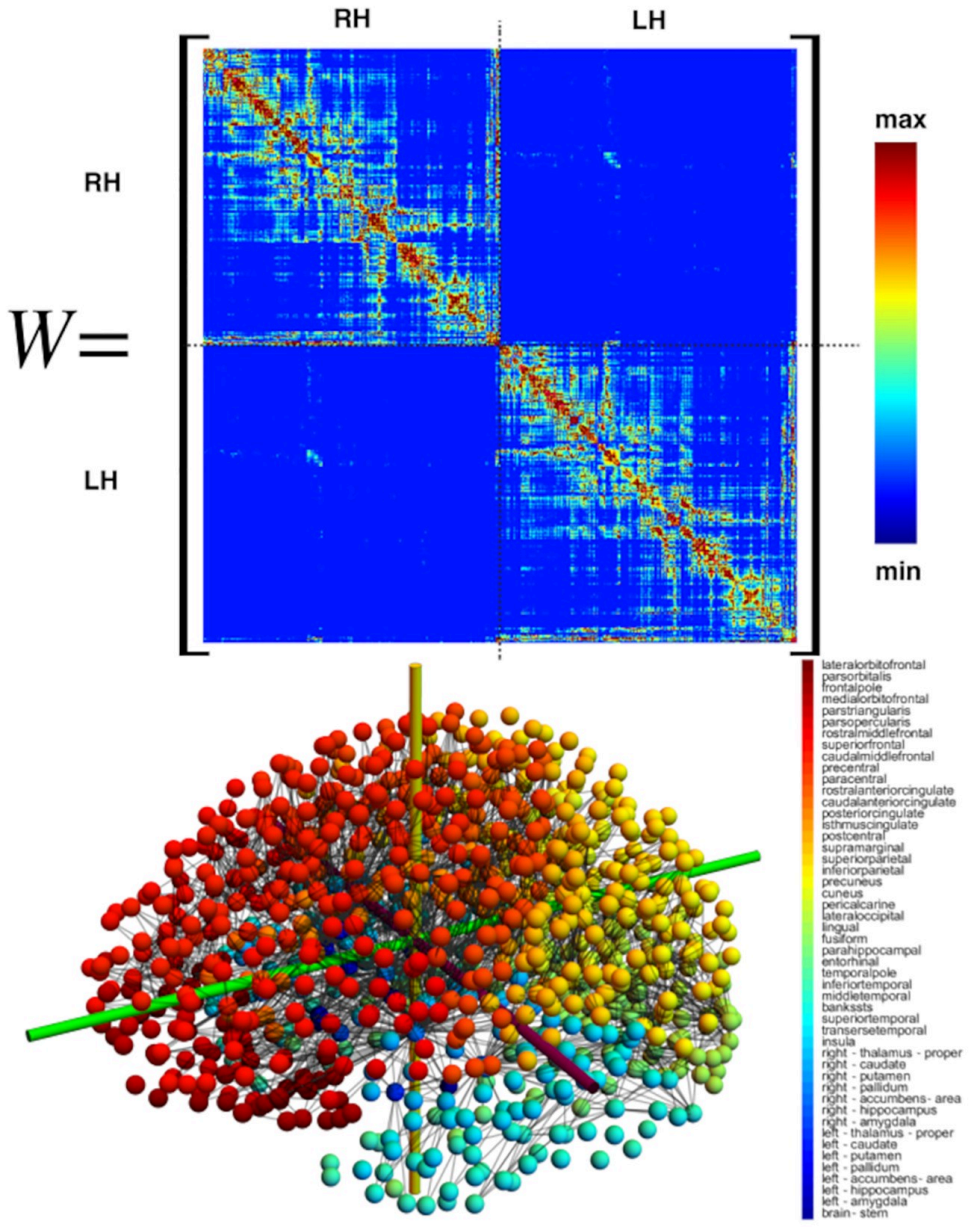
A high-resolution brain structural connectome graph. (Bottom left) The average of 419 brain connectomes with *V* = 1, 015 vertices spanning (bottom right) 49 associated brain regions; the strongest 2,773 edge connections are shown. The weighted adjacency matrix (top) corresponding to the averaged connectome (bottom).

Additionally, we define the graph Laplacian **L** as

$$\begin{array}{c} L_{ij}=\rho \left(D_{ij}-W_{ij}\right),\;i,j=1,\ldots ,V, \end{array}$$ (7)

where *ρ* is an overall effective diffusion constant. The adjacency matrix for the simulation is derived from the tractography of diffusion tensor magnetic resonance images corresponding to 418 healthy subjects of the Human Connectome Project [58] given by Budapest Reference Connectome v3.0 [59]. The graph contains *V* = 1015 nodes and *E* = 70,892 edges and is shown in Fig 21.

Let $\left(u_{j},{\tilde{u}}_{j}\right)$ be the concentration of healthy and toxic A*β* and $\left(v_{j},{\tilde{v}}_{j}\right)$ denote the concentration of healthy and toxic *τ*P at node *j*. The network equations corresponding to the continuous model then take the form of a system of first-order ordinary differential equations. There are four such equations, $\left(u_{j},{\tilde{u}}_{j},v_{j},{\tilde{v}}_{j}\right)$, for each of the 1,015 vertices in the system; these four nodal equations are:

$$\begin{array}{ccc} & & \frac{\text{d}u_{j}}{\text{d}t}=-\sum_{k=1}^{V}L_{jk}u_{k}+a_{0}-a_{1}\;u_{j}-a_{2}\;u_{j}\;{\tilde{u}}_{j}, \end{array}$$ (8)

$$\begin{array}{ccc} & & \frac{\text{d}{\tilde{u}}_{j}}{\text{d}t}=-\sum_{k=1}^{V}L_{jk}{\tilde{u}}_{k}-{\tilde{a}}_{1}\;u_{j}+a_{2}\;u_{j}\;{\tilde{u}}_{j}, \end{array}$$ (9)

$$\begin{array}{ccc} & & \frac{\text{d}v_{j}}{\text{d}t}=-\sum_{k=1}^{V}L_{jk}v_{k}+b_{0}-b_{1}\;v_{j}-b_{2}\;v_{j}\;{\tilde{v}}_{j}-b_{3}\;{\tilde{u}}_{j}\;v_{j}{\tilde{v}}_{j}, \end{array}$$ (10)

$$\begin{array}{ccc} & & \frac{\text{d}{\tilde{v}}_{j}}{\text{d}t}=-\sum_{k=1}^{V}L_{jk}{\tilde{v}}_{k}-{\tilde{b}}_{1}\;{\tilde{v}}_{j}+b_{2}\;v_{j}\;{\tilde{v}}_{j}+b_{3}\;{\tilde{u}}_{j}\;v_{j}{\tilde{v}}_{j}, \end{array}$$ (11)

where *j* = 1, …, *V* = 1, 015. Similarly, for the damage model we define a damage variable *q*_*j*_ at each node *j* and assume the same law

$$\begin{array}{c} {\dot{q}}_{j}=(k_{1}{\tilde{u}}_{j}+k_{2}{\tilde{v}}_{j}+k_{3}{\tilde{u}}_{j}\;{\tilde{v}}_{j}+k_{4}\sum_{k=1}^{V}A_{jk}q_{k})\left(1-q_{j}\right),\;q_{j}\left(0\right)=0,\;j=1,\ldots ,V, \end{array}$$ (12)

where *A*_*jk*_ is the weighted network adjacency matrix *A*_*jk*_ = *n*_*jk*_/*l*_*jk*_ if *j* ≠ *k* (and *n*_*jk*_ > 0) and 0 otherwise. Thus *k*_4_ has the interpretation of a ‘transmission speed’; the time it takes for the effects of degeneracy in cell *k* to reach cell *j*. The weighting chosen in the adjacency matrix term is inspired by the propagation of transneuronal degeneration from a node to its neighbors.

### An analysis of the continuous model

#### Homogeneous system

It is instructive to start with an analysis of the homogeneous system obtained by assuming that there is no spatial dependence. This analysis applies to both network and continuous models. In this case, both systems reduce to the dynamical system

$$\begin{array}{c} \begin{array}{ccc} \frac{\text{d}u}{\text{d}t} & = & a_{0}-a_{1}\;u-a_{2}\;u\;\tilde{u},\\ \frac{\text{d}\tilde{u}}{\text{d}t} & = & -{\tilde{a}}_{1}\;\tilde{u}+a_{2}\;u\;\tilde{u},\\ \frac{\text{d}v}{\text{d}t} & = & b_{0}-b_{1}\;v-b_{2}\;v\;\tilde{v}-b_{3}\;\tilde{u}\;v\;\tilde{v},\\ \frac{\text{d}\tilde{v}}{\text{d}t} & = & -{\tilde{b}}_{1}\;\tilde{v}+b_{2}\;v\;\tilde{v}+b_{3}\;\tilde{u}\;v\;\tilde{v}, \end{array} \end{array}$$ (13)

where all variables and initial conditions are assumed to be positive and all parameters are strictly positive.

#### Damage evolution

For the homogeneous system above the concentrations remain homogeneous for all time. Damage, in contrast, is node-dependent and expressed by the (nodal) variable *q*_*j*_ ∈ [0, 1]. Indeed, in this case, the non-local term associated with transneuronal degeneration, commensurate with the tensor *A*_*jk*_ in Eq (12), cannot be homogeneous. Nevertheless, the damage dynamics are simple enough to describe. Damage will initially increases linearly in time, homogeneously, from the initial value *q*_*j*_ = 0. The increase will then trend exponentially at each node, with node-dependent time scales depending on the local node’s degree, and saturate to the value *q*_*j*_ = 1 asymptotically in time at each node.

#### Stationary points

The stationary points and stability of the homogeneous system (13) are instructive; they inform the disease dynamics implied by the local model. The system (13) can exhibit one, two, three, or four stationary points depending on the parameters; these are:

1. Healthy *τ*P-healthy A*β*: This stationary state is always a solution to (13) and is descriptive of an individual with zero toxic load; no amyloid plaques or neurofibrillary tau tangles. The state is given by:
  $$\begin{array}{c} \left(u_{1},{\tilde{u}}_{1},v_{1},{\tilde{v}}_{1}\right)=(\frac{a_{0}}{a_{1}},0,\frac{b_{0}}{b_{1}},0). \end{array}$$ (14)
2. Healthy *τ*P–toxic A*β*: This state describes a diseased brain wherein some A*β* plaques exist but the tau fibril (NFT) concentration or that of hyperphosphorylated tau is non-existent or negligible. A description of this stationary state in terms of the base problem parameters is:
  $$\begin{array}{c} \left(u_{2},{\tilde{u}}_{2},v_{2},{\tilde{v}}_{2}\right)=(\frac{{\tilde{a}}_{1}}{a_{2}},\frac{a_{0}a_{2}-a_{1}{\tilde{a}}_{1}}{a_{2}{\tilde{a}}_{1}},\frac{b_{0}}{b_{1}},0). \end{array}$$
  In terms of *u*_1_ = *a*_0_/*a*_1_, from (14), and $u_{2}={\tilde{a}}_{1}/a_{2}$ it is given by
  $$\begin{array}{c} \left(u_{2},{\tilde{u}}_{2},v_{2},{\tilde{v}}_{2}\right)=(\frac{{\tilde{a}}_{1}}{a_{2}},\frac{a_{1}\left(u_{1}-u_{2}\right)}{{\tilde{a}}_{1}},\frac{b_{0}}{b_{1}},0). \end{array}$$ (15)
  Since the concentrations must be non-negative: the form of ${\tilde{u}}_{2}$, above, implies that *u*_1_ ≥ *u*_2_. This results in the condition of ${\tilde{a}}_{1}/a_{2}\leq a_{0}/a_{1}$. In other words either the clearance term of toxic A*β* must be sufficiently small, the conversion term must be sufficiently large, or a ratio of the two, to allow for the existence of a toxic state.
3. Toxic *τ*P–healthy A*β*: This stationary state is a conceptual dual to the previous state above; granted, toxic *τ*P does not influence the A*β* population whereas A*β* does induce additional *τ*P formation. As in (15) we express this state, immediately here, in terms of *u*_1_ = *a*_0_/*a*_1_ and *v*_1_ = *b*_0_/*b*_1_ as
  $$\begin{array}{c} \left(u_{3},{\tilde{u}}_{3},v_{3},{\tilde{v}}_{3}\right)=(u_{1},0,\frac{{\tilde{b}}_{1}}{b_{2}},\frac{b_{1}\left(v_{1}-v_{3}\right)}{{\tilde{b}}_{1}}). \end{array}$$ (16)
  Requiring *v*_1_ ≥ *v*_3_ implies that ${\tilde{b}}_{1}/b_{2}\leq b_{0}/b_{1}$.
4. Toxic *τ*P–toxic A*β*: This stationary state reflects the invasion of a patient’s brain by both toxic amyloid beta and toxic tau. As in (14)–(16) we write the state in terms of the previous state variables *u*_1_ = *a*_0_/*a*_1_, $u_{2}={\tilde{a}}_{1}/a_{2}$, ${\tilde{u}}_{2}=a_{1}\left(u_{1}-u_{2}\right)/{\tilde{a}}_{1}$, $v_{3}={\tilde{b}}_{1}/b_{2}$ and *v*_4_, defined below, as:
  $$\begin{array}{c} (u_{4},{\tilde{u}}_{4},v_{4},{\tilde{v}}_{4})=(u_{2},{\tilde{u}}_{2},\frac{a_{2}b_{2}u_{2}v_{3}}{a_{1}b_{3}\left(u_{1}-u_{2}\right)+a_{2}b_{2}u_{2}},\frac{b_{1}{\tilde{a}}_{1}(v_{3}-v_{4})(v_{1}-v_{4})}{a_{1}b_{3}(u_{1}-u_{2})v_{3}v_{4}}). \end{array}$$ (17)
  Introducing
  $$\begin{array}{c} \mu =a_{0}\frac{b_{3}}{b_{2}}, \end{array}$$ (18)
  into (17) gives
  $$\begin{array}{c} (u_{4},{\tilde{u}}_{4},v_{4},{\tilde{v}}_{4})=(u_{2},{\tilde{u}}_{2},\frac{{\tilde{a}}_{1}u_{1}v_{3}}{\mu \left(u_{1}-u_{2}\right)+{\tilde{a}}_{1}u_{1}},\frac{b_{1}{\tilde{a}}_{1}u_{1}(v_{3}-v_{4})(v_{1}-v_{4})}{\mu {\tilde{b}}_{1}(u_{1}-u_{2})v_{4}}). \end{array}$$ (19)

#### Stability

We briefly discuss the stability of the stationary points. In addition we distinguish between the two possible ‘disease’ phenomena of (13): the case of a disease system characterized by the dynamics of a four-stationary-point model and the case of a disease system characterized by three fixed points.

#### Eigenvalues of the linearized system

The linearization of (13) about any fixed point $\left(u,\tilde{u},v,\tilde{v}\right)$ is governed by the Jacobian matrix

$$\begin{array}{c} {}[\begin{array}{cccc} -(a_{2}\tilde{u}+a_{1}) & -a_{2}u & 0 & 0\\ a_{2}\tilde{u} & a_{2}u-{\tilde{a}}_{1} & 0 & 0\\ 0 & -b_{3}v\tilde{v} & -(b_{2}\tilde{v}+b_{1}+b_{3}\tilde{u}\tilde{v}) & -b_{2}v-b_{3}\tilde{u}v\\ 0 & b_{3}v\tilde{v} & b_{2}\tilde{v}+b_{3}\tilde{u}\tilde{v} & b_{2}v-{\tilde{b}}_{1}+b_{3}\tilde{u}v \end{array}]. \end{array}$$ (20)

The first two eigenvalues of (20) correspond to the A*β* subsystem, e.g. $\left(u,\tilde{u}\right)$, of (13). Since the coupling of (13) is a one-way coupling these eigenvalues are given by the corresponding eigenvalues of the uncoupled heterodimer model:

$$\begin{array}{c} \lambda_{A\beta ,1}=-\frac{1}{2}(B+\sqrt{B^{2}-4C}),\;\lambda_{A\beta ,2}=-\frac{1}{2}(B-\sqrt{B^{2}-4C}), \end{array}$$ (21)

where $B\left(u,\tilde{u},a_{1},a_{2},u_{2}\right)=a_{1}+{\tilde{a}}_{1}+a_{2}(\tilde{u}-u)$ and $C\left(u,\tilde{u},a_{1},a_{2},u_{2}\right)=a_{2}({\tilde{a}}_{1}\tilde{u}-a_{1}u)+{\tilde{a}}_{1}a_{1}$. The third and fourth eigenvalues of (20), corresponding to the coupled $\left(v,\tilde{v}\right)$ tau system of (13), can be written as

$$\begin{array}{c} \lambda_{\tau P,1}=-\frac{1}{2}(\hat{B}+\sqrt{{\hat{B}}^{2}-4\hat{C}}),\;\lambda_{\tau P,2}=-\frac{1}{2}(\hat{B}-\sqrt{{\hat{B}}^{2}-4\hat{C}}), \end{array}$$ (22)

with $\hat{B}=B\left(v,\tilde{v},b_{1},{\tilde{b}}_{1},b_{2}\right)+b_{3}\tilde{u}(\tilde{v}-v)$ and $\hat{C}=C\left(v,\tilde{v},b_{1},{\tilde{b}}_{1},b_{2}\right)+b_{3}\tilde{u}\left({\tilde{b}}_{1}\tilde{v}-b_{1}v\right)$. The form of the tau eigenvalues coincides with those for A*β* when *b*_3_ = 0 or when $\tilde{u}$ vanishes.

#### Disease phenomenology

We can interpret the different stationary states in terms of disease dynamics and define, accordingly, different disease states.

#### The healthy brain

A healthy patient represents an instantiation of the healthy stationary state whereby $\tilde{u}=\tilde{v}=0$. For the Healthy *τ*P-healthy A*β* state to exist we must have *a*_0_ ≤ *a*_1_ and *b*_0_ ≤ *b*_1_, i.e., (*u*, *v*) ∈ [0, 1] × [0, 1] are valid concentrations. A failure in healthy clearance, either with an amyloid clearance value satisfying 0 ≤ *a*_1_ < *a*_0_ or with a tau clearance of 0 ≤ *b*_1_ < *b*_0_, implies the non-existence of a physically relevant healthy state (c.f. (14). It is instructive to note that the expressions *a*_0_/*a*_1_ and $a_{2}/{\tilde{a}}_{1}$ (respectively *b*_0_/*b*_1_ and $b_{2}/{\tilde{b}}_{1}$) express a balance of healthy A*β* production to clearance and toxic A*β* production to clearance (respectively healthy *τ*P and toxic *τ*P production to clearance). Consider the following balance of clearance inequalities:

$$\begin{array}{c} \frac{{\tilde{a}}_{1}}{a_{2}}>\frac{a_{0}}{a_{1}},\;\frac{{\tilde{b}}_{1}}{b_{2}}>\frac{b_{0}}{b_{1}}. \end{array}$$ (23)

A patient satisfying (23) enjoys full stability to perturbations while in the healthy state (14). That is: if (23) holds with $\left(u,\tilde{u},v,\tilde{v}\right)$ given by (14) then the real parts of the eigenvalues (21) and (22) are negative and the production of small amounts of toxic A*β*, or of toxic tau, or the excess production of healthy A*β*, or healthy tau, results in a quick return to the healthy homeostatic baseline state of (14). The above implies that the model (8)–(11) recognizes the critical role that clearance plays in neurodegenerative diseases. A low value of toxic clearance ${\tilde{a}}_{1}$, respectfully ${\tilde{b}}_{1}$, with sustained healthy clearance or a low value of healthy clearance *a*_1_, respectfully *b*_1_, with sustained toxic clearance is enough to trigger an instability capable of driving the system away from the healthy state.

#### The susceptible brain

From the previous discussion, we conclude that an unfavorable alteration in clearance mechanisms not only renders the healthy state unstable to perturbations but brings into existence the other stationary points characterizing various pathological conditions.

Indeed, a well established clinical biomarker for Alzheimer’s disease is a drop in soluble amyloid concentration in the cerebrospinal fluid; directly suggesting a decrease in *a*_1_. Recent evidence also suggest [60] that toxic tau filaments in chronic traumatic encephalopathy patients enclose hydrophobic molecules which may contain blood-born pathogens; a possible result of vascular damage from an impact. Such a finding could imply, for instance, that repeated traumatic injury causes vessel rupture and a subsequent proclivity for this unique form of toxic tau production. The stage is then set to trigger a pathological decline when the critical relation (23), corresponding to tau, is violated due to a balance of increased toxic load and age-induced clearance deficit.

The moment of susceptibility occurs when the inequality of (23) becomes an equality. Mathematically, this parameter configuration is a transcritical bifurcation for the homogeneous system (13) at the coincidence of a combination of the states (14)–(16). Clinically, this is the point whereby additional stationary states are physically meaningful and pathology development becomes a possibility.

#### The proteopathic brain

The proteopathic brain has suffered a perturbation from the healthy stationary state; due to the instability in the system this patient is progressing towards a diseased state. The potential pathology phenotypes depend on the patient’s individual parameter values. In particular, if ${\tilde{a}}_{0}/a_{1}\geq a_{1}/a_{2}$ holds then the existence of (15) is physically meaningful and if ${\tilde{b}}_{0}/b_{1}\geq b_{1}/b_{2}$ holds then the same is true of (16). It may be the case, depending on the combination of failed clearance subsystems and specific predisposition for toxic loading, that both relations hold simultaneously. A necessary (clinical) existence criterion for the proteopathic stationary point (17) can be observed directly from the equation for ${\tilde{v}}_{4}$ in (19): namely

$$\begin{array}{c} \mu {\tilde{b}}_{1}\left(u_{1}-u_{2}\right)v_{4}\neq 0. \end{array}$$ (24)

This implies that the parameter *b*_3_, defining *μ* in (18), cannot vanish.

Finally since *b*_3_ ≠ 0 and the numerator of of *v*_4_, in (19), is always non-negative we see that (17) always exists when *u*_1_ > *u*_2_ and when both *v*_3_, *v*_1_ ≥ *v*_4_ or when both *v*_3_, *v*_1_ ≤ *v*_4_. An important observation is that, though the modeling of the pathology of (17) is tied to that of (15) it is not inextricably tied to (16); this is due to the fact that we may always choose *b*_3_, c.f. (18), such *v*_4_ is smaller than both *v*_3_ and *v*_1_. Thus, with a suitably strong A*β* tau-toxification interaction the state (16) is not needed in order to produce tau proteopathy; that is, the model admits a pathology whereby toxic tau is created solely by the presence of toxic A*β*. Therefore, there are two clinically interesting patient proteopathies for our analysis: the case where the patient model consists of all four disease state equilibria, (14)–(17), and the case where the patient model has the three equilibria (14), (15) and (17).

#### Primary tauopathy

**Fig 22:**
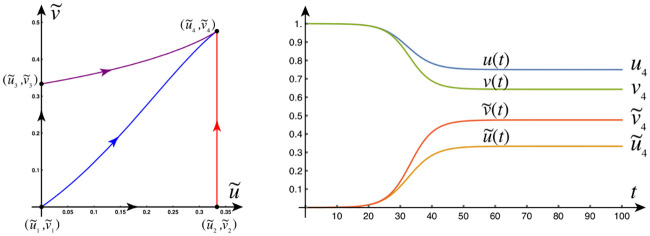
Patient pathology dynamics in primary tauopathy. (Left) Phase plane $\left(\tilde{u},\tilde{v}\right)$ with four equilibria. Homogeneous dynamics of the toxic states. Note that this is a two-dimensional slice of the four-dimensional phase space. (Right) When four different states co-exist, only the fully toxic state is stable as shown by the time-dynamics plot. (Parameters: *a*_0_ = *b*_0_ = *a*_1_ = *a*_2_ = *b*_1_ = *b*_2_ = 1, ${\tilde{a}}_{1}={\tilde{b}}_{1}=3/4$, *b*_3_ = 1/2).

In this case, all four equilibria exist which requires both ${\tilde{a}}_{1}/a_{2}<a_{0}/a_{1}$ and ${\tilde{b}}_{1}/b_{2}<b_{0}/b_{1}$. An example of this dynamic is shown in Fig 22. We see that the presence of toxic A*β* always implies a higher level of *τ* P. Indeed, we have

$$\begin{array}{c} {\tilde{v}}_{4}-{\tilde{v}}_{3}=\frac{a_{1}b_{3}{\tilde{a}}_{1}{\tilde{b}}_{1}^{2}(u_{1}-u_{2})}{b_{2}(a_{1}b_{3}(u_{1}-u_{2})+b_{2}{\tilde{a}}_{1})}>0. \end{array}$$ (25)

We refer to this case as primary tauopathy as the invasion due to *τ*P exists independently of A*β*. The effect of A*β* is to increase the concentration of toxic *τ*P and, possibly, increase the associated damage.

#### Secondary tauopathy

In secondary tauopathy the evolution of *τ*P depends on the primary invasion of A*β*. Parameters corresponding to secondary tauopathy can be obtained by choosing ${\tilde{a}}_{1}/a_{2}<a_{0}/a_{1}$ and ${\tilde{b}}_{1}/b_{2}>b_{0}/b_{1}$ (hence, ${\tilde{v}}_{3}<0$) while taking *b*_3_ large enough so that ${\tilde{v}}_{4}>0$.

**Fig 23:**
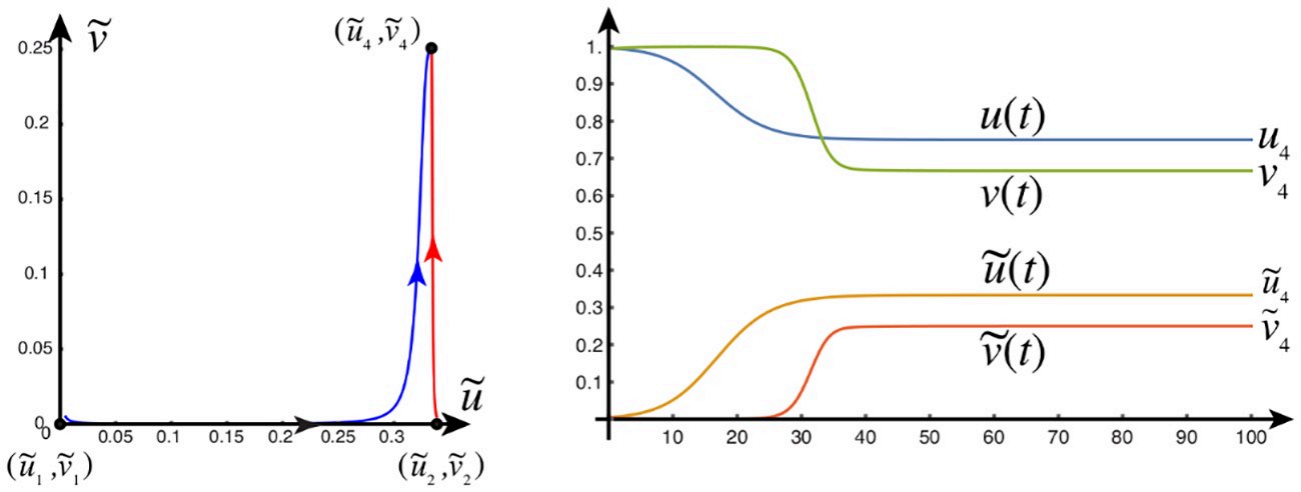
Patient pathology dynamics in secondary tauopathy. (Left) Phase plane $\left(\tilde{u},\tilde{v}\right)$ with three equilibria. (Right) When three different states co-exist, only the fully toxic state is stable as shown by the time-dynamics plot. (Parameters: *a*_0_ = *b*_0_ = *a*_1_ = *a*_2_ = *b*_1_ = *b*_2_ = 1, ${\tilde{a}}_{1}=3/4$, ${\tilde{b}}_{1}=4/3$, *b*_3_ = 3). Note that trajectories are initialized by taking the initial condition *ϵ* = 0.005 away from an equilibrium point.

It is useful to outline key observations, explored further in the manuscript, regarding the nature of the dependence of *τ*P pathology ($\tilde{v}$) on the presence of A*β* pathology ($\tilde{u}$) in this regime. We will see (Front propagation, secondary tauopathy) that the onset of toxic *τ*P follows from the presence of toxic A*β*. We will also see that the speed of toxic *τ*P propagation appears to be limited by the speed of propagation of toxic A*β*. This is distinctly different than the case of primary tauopathy where toxic *τ*P and toxic A*β* can evolve separately. Indeed, Fig 22 (left) shows a stationary point of the form $\left(\tilde{u},\tilde{v}\right)=\left(0,{\tilde{v}}_{3}\right)$ and the fully invaded asymptotic states, Fig 22 (right), satisfy ${\tilde{u}}_{4}<{\tilde{v}}_{4}$; clearly indicating that the additional coupling of $\tilde{u}$ to $\tilde{v}$, in (11) does not limit tau pathology expression, in primary tauopathy, to that of A*β* pathology. In light of the apparent dependence of toxic *τ*P spreading on toxic A*β* propagation in secondary tauopathy it is instructive to enquire whether the asymptotic level of toxic *τ*P pathology concentration be limited by the asymptotic concentration of toxic A*β*? Fig 23 (right) seems to indicate that this is the case.

However, as discussed in the results section (c.f. secondary tauopathy in ‘A simplified model of Alzheimer’s disease proteopathy’), the asymptotic state value of ${\tilde{v}}_{4}$ is not limited by that of ${\tilde{u}}_{4}$. Fig 10a shows that ${\tilde{v}}_{4}>{\tilde{u}}_{4}$ is possible depending on the parameter selection. This observation leads naturally to a further question: is it a strict requirement of secondary tauopathy to have a toxic A*β* concentration at all? That is: can we have $\left({\tilde{u}}_{4},{\tilde{v}}_{4}\right)=\left(0,{\tilde{v}}_{4}\right)$? Suppose this is the case: then, according to (14) and (17), we have ${\tilde{u}}_{4}={\tilde{u}}_{2}=0$ so that *u*_1_ = *u*_2_. When *u*_1_ = *u*_2_ we have, again from (17), that *v*_4_ = *v*_3_; this leaves ${\tilde{v}}_{4}$, in (17), in an indeterminate form. Thus, to understand ${\tilde{v}}_{4}$ when *u*_4_ = 0, we use that $a_{1}=a_{0}u_{1}^{-1}$, c.f. (14), and consider:

$$\begin{array}{c} \lim_{u_{1}\to u_{2}}{\tilde{v}}_{4}=\lim_{u_{1}\to u_{2}}\frac{b_{1}{\tilde{a}}_{1}\left(v_{3}-v_{4}\right)\left(v_{1}-v_{4}\right)}{a_{0}u_{1}^{-1}b_{3}\left(u_{1}-u_{2}\right)v_{3}v_{4}}. \end{array}$$

Due to the indeterminate form of the limit above: we apply L’Hôspital’s rule and compute, after simplifying, that

$$\begin{array}{c} \lim_{u_{1}\to u_{2}}{\tilde{v}}_{4}=\frac{b_{1}\left(v_{1}-v_{3}\right)}{{\tilde{b}}_{1}}. \end{array}$$ (26)

As discussed above: the regime of secondary tauopathy occurs when ${\tilde{v}}_{3}<0$; resulting in (16) being an invalid steady state. This directly implies that *v*_1_ − *v*_3_ < 0 so that the expression (26) is negative. The above shows that if the asymptotic toxic A*β* concentration, ${\tilde{u}}_{4}$, vanishes in the case of secondary tauopathy then the asymptotic toxic tau concentration, ${\tilde{v}}_{4}$ is necessarily negative; this cannot occur in a physical system. To summarize: the sustained presence of a toxic *τ*P population, in secondary tauopathy, requires the presence of toxic A*β*; this implies that the computational observations, for secondary tauopathy, regarding the reliance of toxic *τ*P development and perpetuation, on the presence of A*β* are strict and not merely one possible method of toxic *τ*P development.

#### Front propagation

We can explore the spatio-temporal behavior of the system by first considering a reduction to one dimension (Ω=R) and subsequently analyzing the spread of toxic protein via the study of traveling waves. From the theory of nonlinear parabolic partial differential equations, we expect pulled fronts that connect one equilibrium state to a different homogeneous state [49].

First, consider the two uncoupled fronts emanating from the healthy state $\left(u_{1},{\tilde{u}}_{1},v_{1},{\tilde{v}}_{1}\right)$ and connecting either to $\left(u_{2},{\tilde{u}}_{2},v_{2},{\tilde{v}}_{2}\right)$ or $\left(u_{3},{\tilde{u}}_{3},v_{3},{\tilde{v}}_{3}\right)$. To obtain these fronts, we linearize (3) around the healthy state $\left(u_{1},{\tilde{u}}_{1},v_{1},{\tilde{v}}_{1}\right)$ and obtain the decoupled system

$$\begin{array}{ccc} & & \frac{\partial \tilde{u}}{\partial t}=\left(a_{2}u_{1}-{\tilde{a}}_{1}\right)+{\tilde{d}}_{1}\frac{\partial^{2}\tilde{u}}{\partial x^{2}}, \end{array}$$ (27)

$$\begin{array}{ccc} & & \frac{\partial \tilde{v}}{\partial t}=\;\left(b_{2}v_{1}-{\tilde{b}}_{1}\right)\;+\;{\tilde{d}}_{2}\frac{\partial^{2}\tilde{v}}{\partial x^{2}}. \end{array}$$ (28)

Starting with initial positive data, the system will develop fronts and the asymptotic selected speed is the minimum possible speed for this linear system [61, 62]. Traveling wave solutions to (27) and (28) are obtained explicitly by first performing a traveling wave reduction (*u*(*x*, *t*) → *u*(*z*) with *z* = *x* − *ct* and so on for the other variables) and then looking for linear solutions of the form *u* = *C* exp(λ*z*) which leads to a family of possible solution with speeds *c* = *c*(λ). The smallest such speed is the selected speed for the asymptotic dynamics. In our case, the front speeds are

$$\begin{array}{ccc} & & c_{\beta }^{\left(12\right)}=2\sqrt{{\tilde{d}}_{1}\left(a_{2}a_{0}/a_{1}-{\tilde{a}}_{1}\right)},\;c_{\tau }^{\left(12\right)}=0, \end{array}$$ (29)

where $c_{\beta }^{\left(ij\right)}$ and $c_{\tau }^{\left(ij\right)}$ denote the speeds of the front from state *i* to state *j* (whenever such a front exists) for the A*β* fields $\left(u,\tilde{u}\right)$ and *τ*P fields $\left(v,\tilde{v}\right)$, respectively. The front speeds for the second transition are

$$\begin{array}{ccc} & & c_{\beta }^{\left(13\right)}=0,\;c_{\tau }^{\left(13\right)}=2\sqrt{{\tilde{d}}_{2}\left(b_{2}b_{0}/b_{1}-{\tilde{b}}_{1}\right)}. \end{array}$$ (30)

Similarly, if both fields are seeded initially, we have

$$\begin{array}{ccc} & & c_{\beta }^{\left(14\right)}=c_{\beta }^{\left(12\right)},\;c_{\tau }^{\left(14\right)}=c_{\tau }^{\left(13\right)}. \end{array}$$ (31)

We see that these fronts only exist if $a_{2}a_{0}>{\tilde{a}}_{1}a_{1}$ and/or $b_{2}b_{0}>{\tilde{b}}_{1}b_{1}$ which are the conditions for the existence of toxic states found in the previous section. Trivially, a front between two states can only develop if such states exist.

Second, we consider the possibility of fronts propagating from equilibrium state 2 to state 4. To do so, we linearize the equations around $\left(u_{2},{\tilde{u}}_{2},v_{2},{\tilde{v}}_{2}\right)$ and repeat the previous steps to find

$$\begin{array}{c} c_{\beta }^{\left(24\right)}=0,\;c_{\tau }^{\left(24\right)}=2\sqrt{\frac{{\tilde{\rho }}_{2}}{a_{2}b_{1}{\tilde{a}}_{1}}}\sqrt{{\tilde{a}}_{1}(a_{2}(b_{0}b_{2}-b_{1}{\tilde{b}}_{1})-a_{1}b_{0}b_{3})+a_{0}a_{2}b_{0}b_{3}}. \end{array}$$ (32)

#### Primary tauopathy

**Fig 24:**
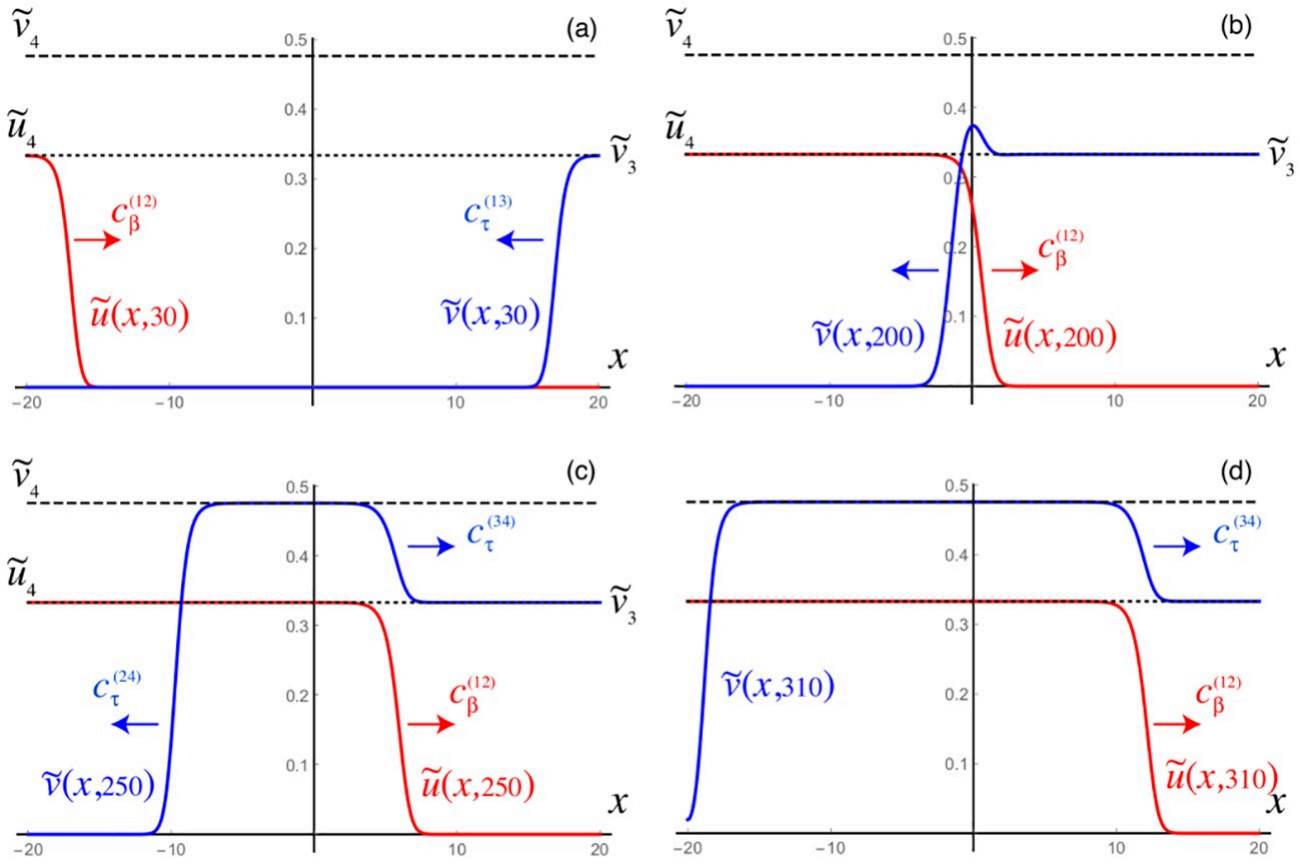
Front dynamics, primary tauopathy.

As an example of the interactions between the two fronts, we consider a toxic A*β* front on the real axis *x* propagating to the right interacting with a *τ*P front propagating to the left (see Fig 24). They evolve initially with constant speeds $c_{\beta }^{\left(12\right)}$ and $c_{\tau }^{\left(13\right)}$ respectively (Fig 24 top). However, when they overlap, the interaction creates an increase in the concentration of *τ*P (Fig 24 top) which both boosts the front to speed $c_{\tau }^{\left(24\right)}>c_{\tau }^{\left(13\right)}$ and initiates a new front propagating backward to fill the interval to the global stable equilibrium ${\tilde{v}}_{4}$ with speed $c_{\tau }^{\left(13\right)}=c_{\beta }^{\left(12\right)}$. The A*β* front is never affected by the presence of toxic *τ*P.

In Fig 24, an A*β* front propagating to the right travels towards a *τ*P front propagating to the left (Fig 24a). The interaction (Fig 24b) increases the toxic level of *τ*P and creates a second front propagating to the right connecting ${\tilde{v}}_{4}$ to ${\tilde{v}}_{3}$ (Fig 24c). The front profiles are shown at time *t* = 30, 200, 250, 310. Parameters are as in Fig 22: *a*_0_ = *b*_0_ = *a*_1_ = *a*_2_ = *b*_1_ = *b*_2_ = 1, ${\tilde{a}}_{1}={\tilde{b}}_{1}=3/4$, *b*_3_ = 1/2, which leads to $c_{\beta }^{\left(12\right)}=c_{\tau }^{\left(13\right)}=c_{\tau }^{\left(34\right)}=0.1$ and $c_{\tau }^{\left(24\right)}=1/\left(2\sqrt{15}\right)\approx 0.13$. Neumann boundary conditions were used on both sides of the finite interval for all variables.

#### Secondary tauopathy

**Fig 25:**
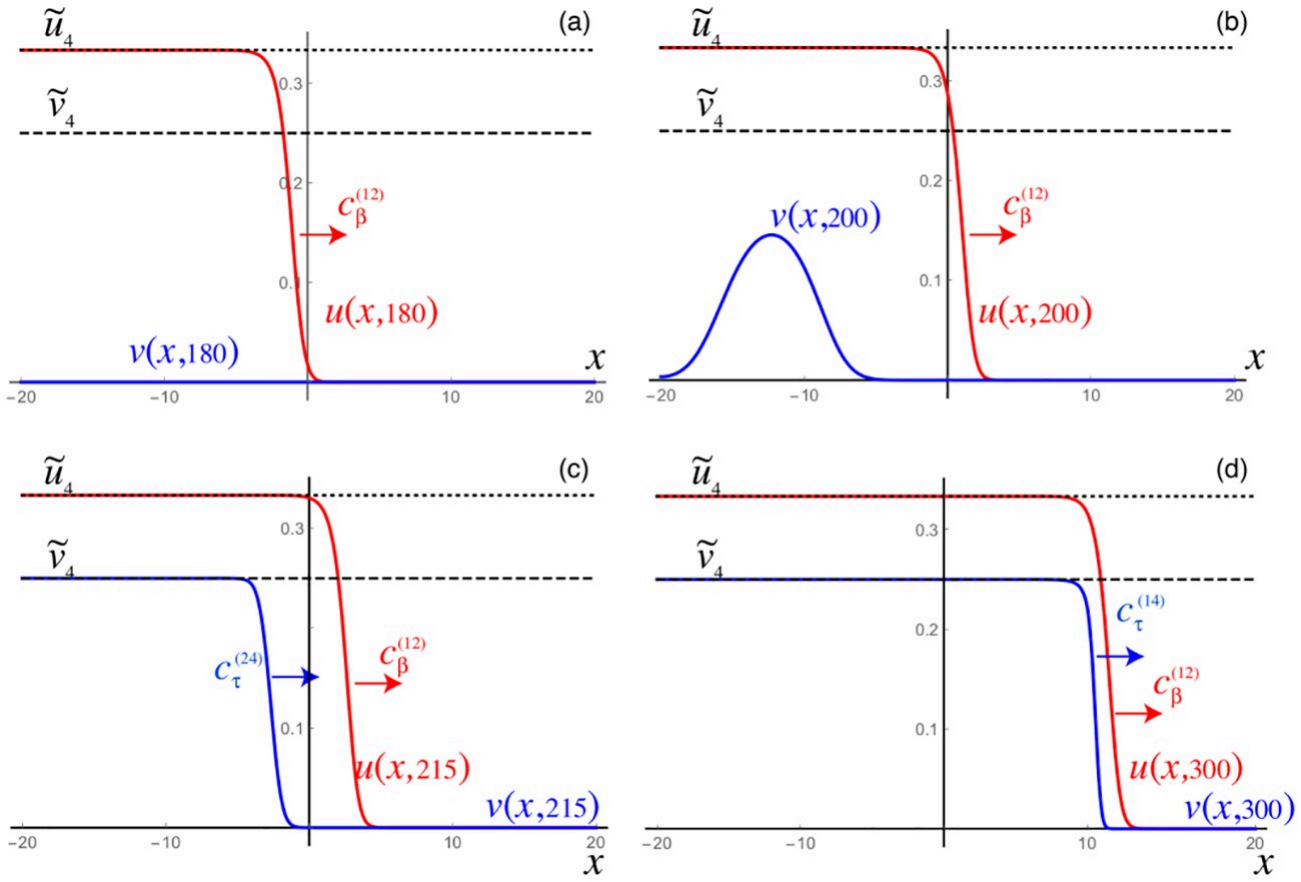
Front dynamics, secondary tauopathy.

As a second example, we consider the case where the A*β* front causes the creation of a non-zero toxic *τ*P state (see Fig 25). Fig 25 depicts the toxic front dynamics of $\tilde{u}\left(x,t\right)$ and $\tilde{v}\left(x,t\right)$; an A*β* front ($\tilde{u}$) propagating to the right in a domain with negligible toxic *τ*P (but with a healthy *τ* population). The passage of the front leads to the rapid expansion of toxic *τ*P (Fig 25b) which evolves at a speed close to $c_{\tau }^{\left(23\right)}>c_{\beta }^{\left(12\right)}$ (Fig 25c). Hence, it eventually catches up with the front (d) and matches its speed. The front profiles are shown at time *t* = 180, 200, 215, 300. Parameters as in Fig 23: *a*_0_ = *b*_0_ = *a*_1_ = *a*_2_ = *b*_1_ = *b*_2_ = 1, ${\tilde{a}}_{1}=3/4$, ${\tilde{b}}_{1}=4/3$, *b*_3_ = 3, which leads to $c_{\beta }^{\left(12\right)}=c_{\tau }^{\left(13\right)}=0.1$ and $c_{\tau }^{\left(24\right)}=\sqrt{2/3}/5\approx 0.1633$. Homogeneous Neumann boundary conditions are used on both sides of the finite interval for all variables. Initial seeding of toxic *τ*P on the positive interval only with $\tilde{v}\left(x,0\right)=10^{-11}$ for *x* > 0 and 0 otherwise.

Finally, the front propagating from equilibrium state 3 to state 4 is constrained by the evolution of the *u* and $\tilde{u}$ fields. Therefore, we find

$$\begin{array}{c} c_{\beta }^{\left(34\right)}=c_{\tau }^{\left(34\right)}=c_{\beta }^{\left(12\right)}. \end{array}$$ (33)

## Supporting information

S1 Video Front dynamics in primary tauopathy, synthetic. Visualization of an illustrative primary tauopathy model simulation using a synthetic channel domain.
(MP4)

S2 Video Front dynamics in primary tauopathy, connectome. Visualization of an illustrative primary tauopathy model simulation using a high-resolution structural brain connectome domain.
(MP4)

S3 Video Front dynamics in secondary tauopathy, synthetic. Visualization of an illustrative secondary tauopathy model simulation using a synthetic channel domain.
(MP4)

S4 Video Front dynamics in secondary tauopathy, connectome. Visualization of an illustrative secondary tauopathy model simulation using a high-resolution structural brain connectome domain.
(MP4)

S5 Video Toxic A*β* in primary and secondary tauopathy. Visualization of the toxic A*β* species in an illustrative model simulation using a high-resolution structural brain connectome domain. Qualitative propagation of the A*β* species was identical in both primary and secondary tauopathy.
(MP4)

S6 Video Toxic *τ*P in primary tauopathy, connectome. Visualization of the toxic *τ*P species in an illustrative model simulation of primary tauopathy using a high-resolution structural brain connectome domain.
(MP4)

S7 Video Toxic *τ*P in secondary tauopathy, connectome. Visualization of the toxic *τ*P species in an illustrative model simulation of secondary tauopathy using a high-resolution structural brain connectome domain.
(MP4)

S1 Data Structural brain connectome, low-resolution. A low-resolution graph of the structural brain connectome. The graph is expressed in a standard format (graphml) based on the human-readable XML markup language. This graph consists of 83 vertices (anatomical regions of interest) and 1,654 edges; data for the graph was sourced from freely-available patient connectome data (https://braingraph.org).
(GRAPHML)

S2 Data Structural brain connectome, high-resolution. A high-resolution graph of the structural brain connectome. The graph is expressed in a standard format (graphml) based on the human-readable XML markup language. This graph consists of 1,015 vertices (anatomical regions of interest) and 70,892 edges; data for the graph was sourced from freely-available patient connectome data (https://braingraph.org).
(GRAPHML)

S1 Appendix Additional detail and solver verification. This appendix contains additional detail regarding the expected behaviour of the network dynamics for specific choices of synthetic parameters. This appendix refers to the equations, and figures, in the main manuscript and is intended to provide a set of verification cases for use in implementing solvers.
(PDF)
