# Supplementary material for: Protein-protein interactions in neurodegenerative diseases: A conspiracy theory
Source: PLoS Comput Biol. 2020 Oct 13;16(10):e1008267. doi: 10.1371/journal.pcbi.1008267 (PMC7584458; doi:10.1371/journal.pcbi.1008267)
Supplement: S1 Appendix — This appendix contains additional detail regarding the expected behaviour of the network dynamics for specific choices of synthetic parameters. This appendix refers to the equations, and figures, in the main manuscript and is intended to provide a set of verification cases for use in implementing solvers. (PDF) [file pcbi.1008267.s010.pdf]

Supplementary Appendix

This supplementary text is intended to assist in the development of a solver for the system of ordinary equations (8)-(12). Here, verification cases are discussed that can be used to compare your solver against; if you use the indicated values then you should produce the exact results as discussed. This supplementary text refers to sections, equation numbers, and figures within the main text and should be use alongside it.

Numerical verification

In this appendix we test our computational platform by recovering the basic homogeneous dynamics of the full network model. To do this we use two hypothetical sets of illustrative, non-clinical parameters; one set of parameters for each regime. We will illustrate the four possible patient states (stationary points) discussed in the Methods section (An Analysis of the continuous model). In Section the primary and secondary tauopathy (Methods, Stability) patient state transitions are simulated and model patient dynamics are discussed in more detail. Front propagation in the brain connectome network is confirmed using synthetic left-right hemisphere initial seedings as discussed in the results section.

Patient states of the network system

We now briefly illustrate the four stationary states of the homogeneous system (Methods, An Analysis of the continuous model). To demonstrate that each of the predicted stationary points is indeed a stationary point of the homogeneous network system, we select illustrative parameters that satisfy the requisite characterizing inequalities. Every node in the brain network is then seeded with the initial value corresponding to the selected fixed point. We expect, and demonstrate, that the system remains stable at that fixed point.

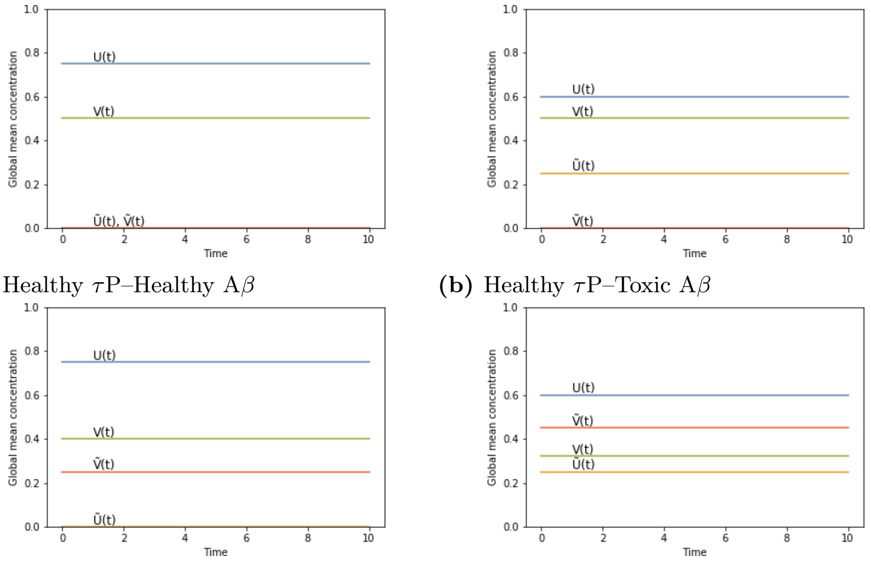

(a) Healthy τP–Healthy Aβ **(b)** Healthy τP–Toxic Aβ

(c) Toxic τP–Healthy Aβ **(d)** Toxic τP–Toxic Aβ

Fig 26. Computational verification of the stationary points (14)-(17)

We will confirm the stationary points by selecting the effective diffusion constant, ρ

of (7), as unity and solving (8)-(12) for $t \in [0, 10]$ using one thousand time-steps. For the healthy Aβ-healthy τP state, c.f. (14), we select $a_0 = 0.75$ and $b_0 = 0.5$; all other parameters are set to unity. All nodes were seeded with the corresponding initial value

$$(u_1, \tilde{u}_1, v_1, \tilde{v}_1) = (\frac{a_0}{a_1}, 0, \frac{b_0}{b_1}, 0) = (0.75, 0, 0.5, 0). \tag{34}$$

Figure 26a shows the plot of global mean tracer concentration with time and confirms that the healthy Aβ-healthy τP state is stationary under the given conditions. For the healthy τP-toxic Aβ fixed point, c.f. (15), we begin with the previous parameters and reduce the toxic Aβ clearance by 40%. We therefore have $\tilde{a}_1 = 0.6$ and keep the previous parameters fixed. We then have

$$(u_2, \tilde{u}_2, v_2, \tilde{v}_2) = (\frac{\tilde{a}_1}{a_2}, \frac{a_1}{\tilde{a}_1}\left(\frac{a_0}{a_1} - \frac{\tilde{a}_1}{a_2}\right), \frac{b_0}{b_1}, 0) = (0.6, 0.25, 0.5, 0).$$

The stationary behavior is again demonstrated; c.f. Figure 26b. For the third stationary state, given by (16), we begin once more with the parameters of the healthy Aβ-healthy τP state and reduce the toxic tau clearance parameter by 60%. We then have $\tilde{b}_1 = 0.4$ and keep all other parameters as in the healthy Aβ–healthy τP state. All nodes are then set to the corresponding initial value

$$(u_3, \tilde{u}_3, v_3, \tilde{v}_3) = (\frac{a_0}{a_1}, 0, \frac{\tilde{b}_1}{b_2}, \frac{b_1}{\tilde{b}_1}\left(\frac{b_0}{b_1} - \frac{\tilde{b}_1}{b_2}\right)) = (0.75, 0.0, 0.4, 0.25).$$

Once more, Figure 26c, we see the stationary characteristic we expect. For the final stationary point, c.f. (19), we use the reduced toxic clearance parameters from the second and third stationary points above, $\tilde{a}_1 = 0.6$ and $\tilde{b}_1 = 0.4$, in addition to the original production values, $a_0 = 0.75$ and $b_0 = 0.5$, of Aβ and τP respectively. All other parameters not explicitly mentioned are again taken to be unity. Given these choices we can directly compute μ and v_4, via (18)-(19), as

$$\mu = a_0 \frac{b_3}{b_2} = 0.75, \quad \text{and} \quad v_4 = \frac{a_0 \tilde{b}_1 \tilde{a}_1}{a_1 b_2}\left(\mu\left(\frac{a_0}{a_1} - \frac{\tilde{a}_1}{a_2}\right) + \tilde{a}_1 \frac{a_0}{a_1}\right)^{-1} = 0.32.$$

Using the above, along with the expressions for v_1, v_3, u_1, u_2 and $\tilde{u}_2$ from (14)-(16), the value of $\tilde{v}_4$ is given directly from the fourth entry of (19) as

$$(u_4, \tilde{u}_4, v_4, \tilde{v}_4) = (u_2, \tilde{u}_2, v_4, \tilde{v}_4) = (0.6, 0.25, 0.32, 0.45).$$

The final plot, for the fourth stationary point, is shown in Figure 26d. Coronal and sagittal plane views of the stationary point verification computation at $t = 10$ are shown in Figure 27.

Patient pathology transitions of the network system

We briefly illustrate the homogeneous state dynamics of the network system; verifying the theoretical view of advanced in the Methods section (Methods, Stability and Disease Phenomenology) on the complex brain network geometry of Figure 21.

Primary tauopathy

We consider a hypothetical susceptible model patient characterized by the parameters previously chosen (S1 Appendix, Patient states of the network system). All four of the stationary points (Methods, An Analysis of the continuous model) coexist with this

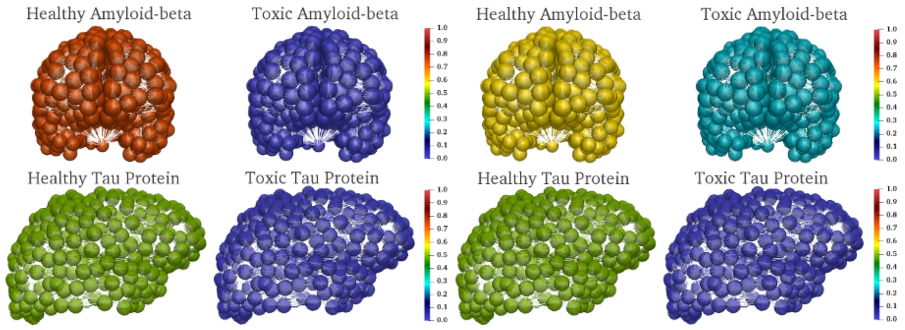

(a) Healthy τP–Healthy Aβ **(b)** Healthy τP–Toxic Aβ

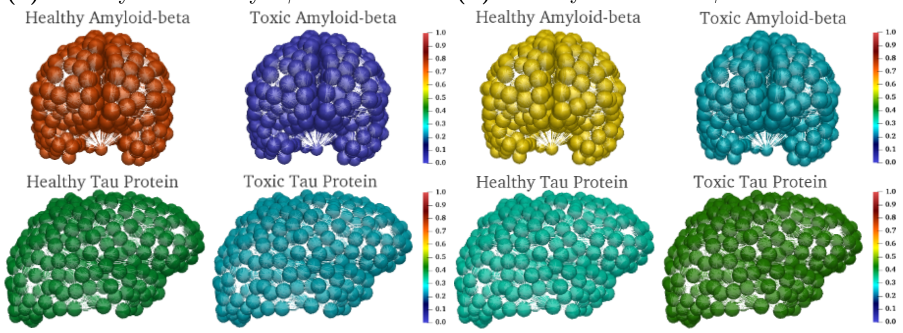

(c) Toxic τP–Healthy Aβ **(d)** Toxic τP–Toxic Aβ

Fig 27. Network system stationary point realization; coronal (top) and sagittal (bottom) views.

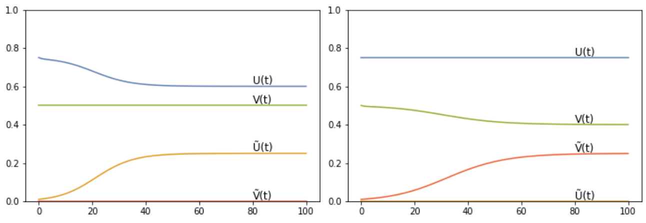

(a) HτP–HAβ to HτP–TAβ **(b)** HτP–HAβ to TτP–HAβ

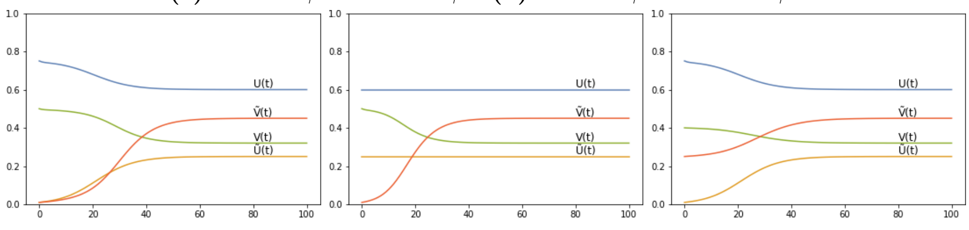

(c) HτP–HAβ to TτP–TAβ **(d)** HτP–TAβ to TτP–TAβ **(e)** TτP–HAβ to TτP–TAβ

Fig 28. State transitions in primary tauopathy. Concentration (y axis) vs. simulation time.

choice of parameters; hence, these parameters fall into the regime of primary tauopathy. In this section we verify the homogeneous state transitions, between the states of Figure 27, of (8)-(12) discretized on the brain network geometry of Figure 21. The selected illustrative primary tauopathy parameters are collected in Table 1 for posterity.

The eigenvalues, (21) and (22), at the healthy Aβ–healthy τP stationary point $(u, \tilde{u}, v, \tilde{v}) = (0.75, 0, 0.5, 0)$ can be calculated. We see that $\lambda_{A\beta,1}, \lambda_{\tau P,1} < 0$, i.e. stable to healthy Aβ and τP perturbations, while $\lambda_{A\beta,2}, \lambda_{\tau P,2} > 0$ so that the otherwise healthy patient brain is susceptible to perturbations in both toxic Aβ and toxic τP. Utilizing the given parameters to evaluate the stability properties at the second stationary point, $(u, \tilde{u}, v, \tilde{v}) = (0.6, 0.25, 0.5, 0)$ c.f. (15), we have $\lambda_{A\beta,1}, \lambda_{A\beta,2}, \lambda_{\tau P,1} < 0$ and $\lambda_{\tau P,2} > 0$; at this state the patient is susceptible only to a perturbation in toxic tau. Likewise at the third stationary point, $(u, \tilde{u}, v, \tilde{v}) = (0.75, 0, 0.4, 0.25)$ c.f. (16), we have $\lambda_{A\beta,1}, \lambda_{\tau P,1}, \lambda_{\tau P,2} < 0$ and $\lambda_{A\beta,2} > 0$ so that the patient in this state is only susceptible to an addition of toxic Aβ. Finally the fixed point (17) is fully stable, i.e. all eigenvalues are negative, and no further disease transition is possible from this state.

Verifications of the primary tauopathy homogeneous state transitions, first depicted in Figure 22, for the full connectome simulation are shown in Figure 28. For instance the healthy state, $(u_1, \tilde{u}_1, v_1, \tilde{v}_1)$, perturbation with respect to both toxic Aβ and toxic τP results in the fully toxic state, $(u_4, \tilde{u}_4, v_4, \tilde{v}_4)$; this is shown in Figure 28c and appears in Figure 22 as the blue (diagonal) path.

Secondary tauopathy

The secondary tauopathy disease model arises when $v_1 < v_3$, so that the stationary point (16) is in an unphysical state, while (14), (15) and (17) remain well defined. One

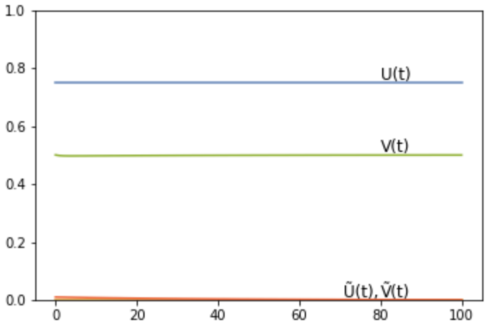

Fig 29. HτP-HAβ, $\tilde{v}$ stable

way that this can be achieved is for b_3, the coefficient mediating the effect of toxic Aβ protein on inducing healthy tau toxification, to be such that both $v_4 < v_1$ and $v_4 < v_3$; a decrease in b_2 can also accomplish this goal, c.f. (17).

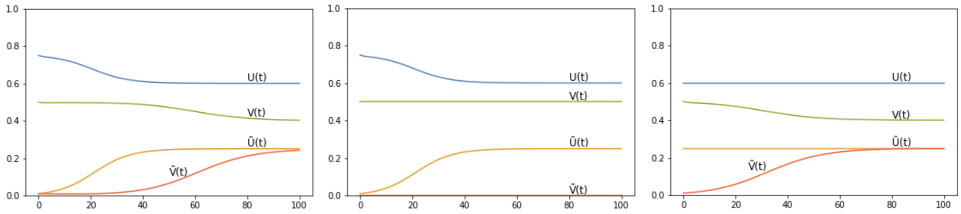

(a) HτP–HAβ to TτP–TAβ **(b)** HτP–HAβ to HτP–TAβ **(c)** HτP–TAβ to TτP–TAβ

Fig 30. State transitions in secondary tauopathy. Concentration vs. simulation time.

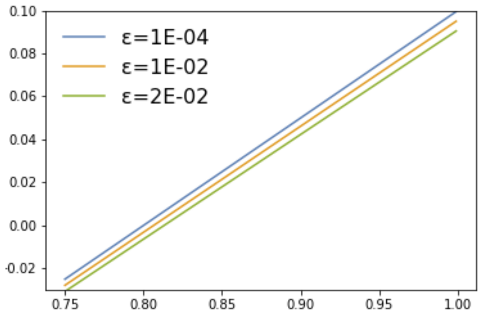

Fig 31. $\lambda_{\tau P,2}$ vs. $\theta = \theta(\tilde{u})$

The condition $v_1 < v_3$ is equivalent to $b_0 b_2 < \tilde{b}_1 b_1$. One can transform the primary tauopathy patient described by the parameters of Table 1 to a secondary tauopathy patient by decreasing b_2 by twenty-five percent; from 1.0 to 0.75. In this regime $v_1 = 0.5$ and $v_3 = 0.5\bar{3}$ and the stationary point (16) is physically inadmissible. The admissible stationary states are

$$(u_1, \tilde{u}_1, v_1 \tilde{v}_1) = (0.75, 0.0, 0.5, 0.0),$$
$$(u_2, \tilde{u}_2, v_2 \tilde{v}_2) = (0.6, 0.25, 0.5, 0.0), \quad (u_4, \tilde{u}_4, v_4 \tilde{v}_4) = (0.6, 0.25, 0.4, 0.25).$$

We see that the first and second stationary points are identical to the case of primary tauopathy and the fourth is perturbed in the $(v, \tilde{v})$ components. Strictly speaking, the healthy patient in this regime is susceptible only to toxic Aβ infection; that is $\lambda_{A\beta,1}, \lambda_{\tau P,1}, \lambda_{\tau P,2} < 0$ and $\lambda_{A\beta,2} > 0$ at $(u_1, \tilde{u}_1, v_1, \tilde{v}_1)$. Verification of the healthy state robustness to perturbations in toxic tau, $\tilde{v}$, is shown in Figure 29.

At the healthy state $\lambda_{A\beta,2} > 0$ holds. Thus, the susceptible, but otherwise healthy, secondary tauopathy patient is at risk of directly developing Aβ proteopathy. This is verified by perturbing the healthy state by a small concentration in $\tilde{u}$; the pursuant transition from the Healthy τP–Healthy Aβ state to the Healthy τP–Toxic Aβ state is pictured in Figure 30b. Having arrived at $(u_2, \tilde{u}_2, v_2, \tilde{v}_2)$ the patient is now susceptible to tauopathy as $\lambda_{\tau P,2} > 0$ there; perturbing $\tilde{v}$ then develops to the Toxic τP–Toxic Aβ state as shown in Figure 30c.

In fact, as postulated, (Results, Stability and Disease Phenomenology Figure 23) the fully diseased state $(u_4, \tilde{u}_4, v_4, \tilde{v}_4)$ is reachable from the healthy state provided that toxic Aβ is present alongside some toxic tau perturbation. This can be seen directly from $\lambda_{\tau P,2}$ in (22). Consider the Taylor expansion of (22), evaluated with $b_2 = 0.75$ and all other parameters as in Table 1, about $\tilde{v} = 0$. We first set $\theta = \tilde{u} + 0.6$ and we let $0 \leq \epsilon \ll 1$ be denote a small perturbation in $\tilde{v}$. It is evident that the effect on $\lambda_{\tau P,2}$ due to a perturbation in toxic tau depends here on both toxic amyloid, $\tilde{u}$, and healthy tau, v, concentration levels. Then, using that $\tilde{u} \geq 0$, and $v \geq 0$, we approximate (22), to order ϵ^2, around $\tilde{v} = 0$ by

$$\lambda_{\tau P,2}(\epsilon) \approx \theta v \left(1 - \frac{\epsilon\theta}{\theta v + 0.6}\right) - 0.4. \tag{35}$$

If we presume, for instance, that the susceptible secondary tauopathy patient has healthy levels of tau protein, i.e. that $v = v_1 = 0.5$, we can directly visualize the effect of toxic Aβ on $\lambda_{\tau P,2}$. Figure 31 shows the approximate value of $\lambda_{\tau P,2}$ (y-axis, c.f. (35)) versus the toxic Aβ value $\theta(\tilde{u}) = \tilde{u} + 0.75$ (x-axis) for three given perturbations ϵ. Evidently, as ϵ decreases the effect of $\tilde{u}$ on increasing $\lambda_{\tau P,2}$ is not diminished. Thus an initial toxic τP seed will develop into a full blown infection provided $\tilde{u}$ is present, or quickly develops, in sufficient quantity to evolve $\lambda_{\tau P,2}$ above zero. This is precisely the

predicted behavior (Results, Figure 23). In accordance we see, c.f. Figure 30a, that perturbing both $\tilde{u}$ and $\tilde{v}$ simultaneously from the initial healthy state induces direct evolution to fully diseased state.
